# Are U–U Bonds Inside Fullerenes Really Unwilling Bonds?

**DOI:** 10.1021/jacs.2c12346

**Published:** 2023-03-06

**Authors:** Antonio Moreno-Vicente, Yannick Roselló, Ning Chen, Luis Echegoyen, Paul W. Dunk, Antonio Rodríguez-Fortea, Coen de Graaf, Josep M. Poblet

**Affiliations:** † Departament de Química Física i Inorgànica, Universitat Rovira i Virgili, Marcel·lí Domingo 1, Tarragona 43007, Spain; ‡ Laboratory of Advanced Optoelectronic Materials, College of Chemistry, Chemical Engineering and Materials Science, Soochow University, Suzhou, Jiangsu 215123, P. R. China; § Department of Chemistry, University of Texas at El Paso, 500 West University Avenue, El Paso, Texas 79968, United States; ∥ Ion Cyclotron Resonance Program, National High Magnetic Field Laboratory, Florida State University, Tallahassee, Florida 32310, United States; ⊥ ICREA, Pg. Lluis Companys 23, Barcelona 08010, Spain

## Abstract

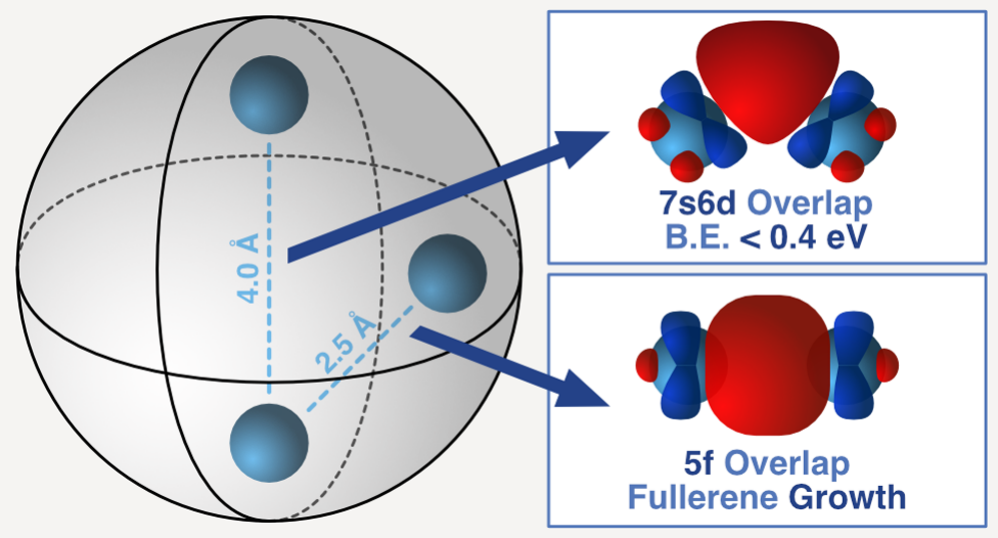

Previous characterizations of diactinide endohedral metallofullerenes (EMFs) Th_2_@C_80_ and U_2_@C_80_ have shown that although the two Th^3+^ ions form a strong covalent bond within the carbon cage, the interaction between the U^3+^ ions is weaker and described as an “unwilling” bond. To evaluate the feasibility of covalent U–U bonds, which are neglected in classical actinide chemistry, we have first investigated the formation of smaller diuranium EMFs by laser ablation using mass spectrometric detection of dimetallic U_2_@C_2n_ species with 2*n* ≥ 50. DFT, CASPT2 calculations, and MD simulations for several fullerenes of different sizes and symmetries showed that thanks to the formation of strong U(5f^3^)-U(5f^3^) triple bonds, two U^3+^ ions can be incarcerated inside the fullerene. The formation of U–U bonds competes with U–cage interactions that tend to separate the U ions, hindering the observation of short U–U distances in the crystalline structures of diuranium endofullerenes as in U_2_@C_80_. Smaller cages like C_60_ exhibit the two interactions, and a strong triple U–U bond with an effective bond order higher than 2 is observed. Although 5f–5f interactions are responsible for the covalent interactions at distances close to 2.5 Å, overlap between 7s6d orbitals is still detected above 4 Å. In general, metal ions within fullerenes should be regarded as templates in cage formation, not as statistically confined units that have little chance of being observed.

## Introduction

Understanding how atoms stay together to form stable structures is at the core of chemistry.^1−3^ In nature, uranium is generally found as an oxide, showing several oxidation states.^4^ Huge advances have been made in the chemistry of the f-elements over the last decades,^5,6^ including the synthesis of complexes containing actinide–ligand multiple bonds,^7^ or uranyl peroxide capsules with high oxidation states.^8−10^ Nevertheless, much less experimental information is available about the formation of diactinides. Since the initial studies, it became clear that the propensity to form U-ligand bonds was greater than that for forming U–U bonds.^11^ Mass spectrometric evidence has been reported on the formation of Th_2_ and U_2_ in gas phase^12^ as well as the isolation and characterization of uranium hydride molecules.^13^ The complexity of the electronic structure of the naked U_2_ was already pointed out in 1990,^1,14^ but it was not until 2005 when Gagliardi and Roos predicted using CASPT2 calculations that U_2_ is a stable molecule in the gas phase with a dissociation energy of about 30 kcal·mol^–1^.^15^ The computed U–U bond distance of 2.43 Å was interpreted in terms of the presence of a quintuple bond. Since that seminal work, several theoretical articles have been devoted to the analysis and rationalization of the U–U interaction in different environments.^16−19^ Using state-of-the-art relativistic quantum chemical methods, Knecht, Jensen and Saue reinterpreted recently the nature of the bond in U_2_, proposing that the U–U interaction corresponds to a quadruple bond, with an internuclear distance of 2.56 Å and a dissociation energy higher than 21 kcal·mol^–1^.^20^

Since the pioneering article in which Sc_3_N was captured within a C_80_ cage,^21^ many endohedral fullerenes have been synthesized and characterized,^22,23^ including actinide endofullerenes,^24−27^ where Th and U tend to act as tetravalent or trivalent ions. Recently, our groups synthesized and characterized for the first time a dimetallic actinide endofullerene, U_2_@C_80_.^28^ Like for previous dimetallic lanthanides, La_2_@C_80_^29^ and Ce_2_@C_80_,^30^ the metal–metal distance was found to be very long. The X-ray diffraction data revealed that the uranium–uranium separations ranged between 3.46 and 3.79 Å. This situation was previously predicted from computations by Straka and co-workers and interpreted as a result of the ion–ion repulsions and metal–cage interactions.^31^ The interaction between the two U atoms was described as an unwilling bond. The concept of strong ion repulsion often used to describe endohedral metallofullerenes^32^ is somewhat contradictory with the experimentally observed formation of dimetallic or even trimetallic species such as Y_3_@C_80_,^33^ inside relatively small empty cavities. Very recently, our teams synthesized and characterized a new endofullerene with two Th atoms.^34^ Within the *I*_h_-C_80_ fullerene, the two Th atoms exhibit formal charges of +3 with a long Th–Th distance of 3.82 Å. In contrast to the diuranium endofullerene, the Th–Th interaction was characterized as a *strong covalent* bond between two actinides, its strength estimated to be greater than 40 kcal·mol^–1^. In organometallic chemistry, the metal–metal interactions with participation of f-block elements have attracted the interest of experimentalists and theoreticians.^35^ Numerous compounds have been reported containing metal–metal bonds between a Ln (or An) and a TM, and also between two Ln ions. In some bimetallic clusters with An–TM bonds, the presence of a weak direct U–U interaction has been proposed, as occurs in recently reported systems with four (or six) U–Ni bonds.^36^ DFT calculations also suggest that for [{Th(η^8^–C_8_H_8_)(μ_3_–Cl)_2_}_3_{K(THF)_2_}_2_], the three-thorium cluster has a delocalized three-center-two-electron Th–Th bond.^37^ In all of these cases, the An–An interaction is assumed to be very weak.

In the early work of Smalley and co-workers, it was already shown that in addition to several small and medium-sized mono-uranium endofullerenes,^38,39^ small diuranium endo-fullerenes, such as U_2_@C_58_ and U_2_@C_52_, could also be detected by mass spectrometry. Without a strong metal–metal interaction, it is very difficult to understand that such dimetallic fullerenes can be formed and grow at the very high temperatures at which these compounds are synthesized.^40,41^ In the present paper, we report Car–Parrinello MD simulations in combination with DFT and CASPT2 calculations to evaluate under which conditions, if any, the formation of covalent bonds between two U ions within fullerenes is possible. To assess the potential role that metal–metal bonds may play during the formation of the endofullerenes, we have resynthesized U_2_@C_2*n*_ species in the gas phase by ablation of uranium-doped graphite and have identified the *smallest* diuranium endofullerenes that are formed, trying to understand the limits to encapsulate two U atoms inside an empty carbon cage.

## Results and Discussion

### Gas-Phase Synthesis of Small Endohedral Diuranium Fullerenes from Doped Graphite

**Figure 1:**
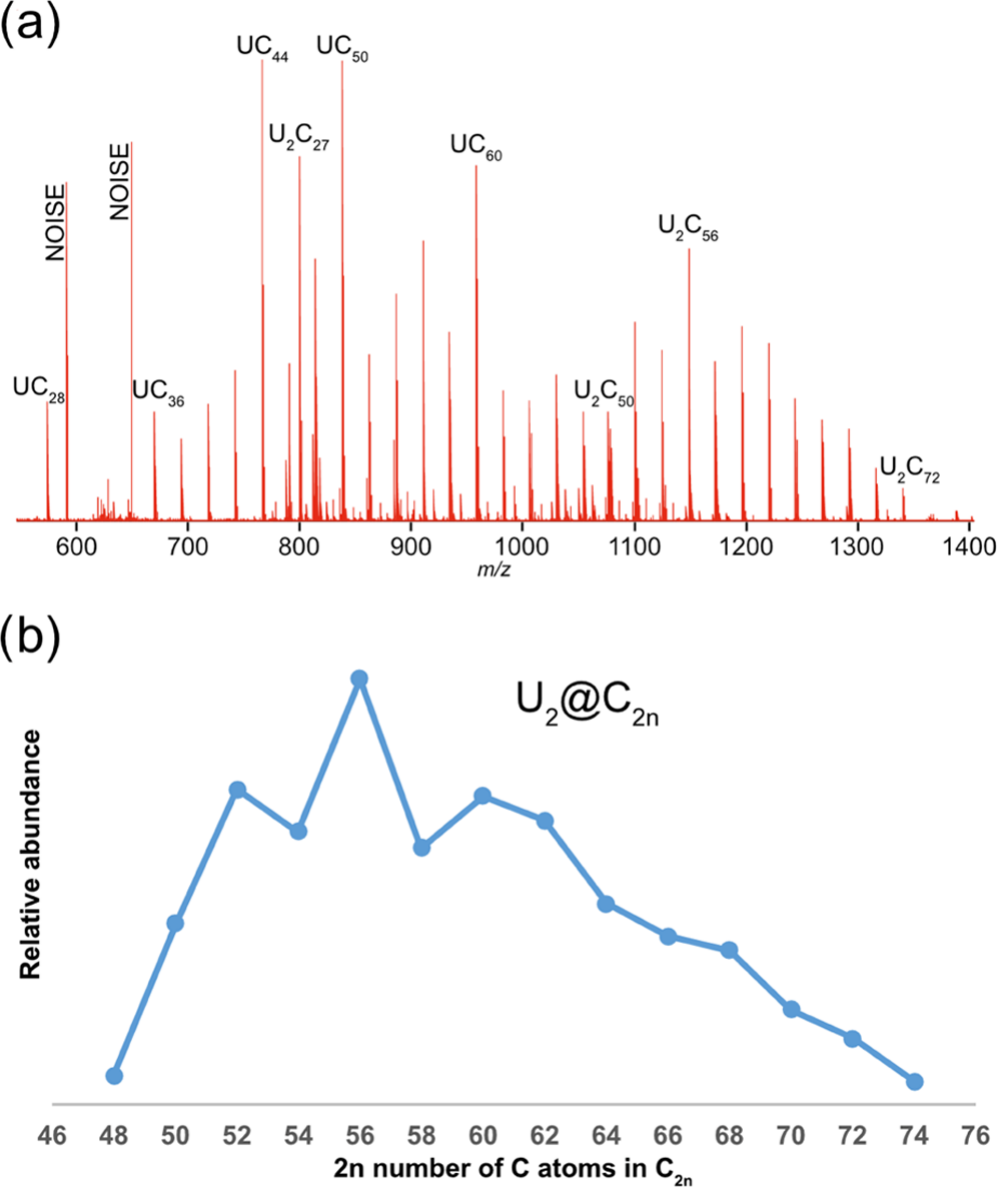
(a) FT-ICR mass spectrum of cluster cations formed from laser ablation of graphite doped with 10% of uranium. Some characteristic peaks for mono- and diuranium compounds are assigned (see also Figure S2). (b) Relative abundances for diuranium U_2_@C_2*n*_ species obtained in gas phase.

Dunk et al. reported that small, medium, and large endohedral mono-metallofullerenes are formed in the gas phase by laser vaporization of metal-doped graphite through a bottom-up mechanism.^39,40^ In this mechanism, it is assumed that the smallest EMF compounds for a given element are the first to form. Hence, for example, the smallest fullerene was identified as an M@C_28_, with M = Ti, Zr, U, where the highly strained carbon cage is stabilized by a four-electron transfer from the metal to the cage and by metal–cage interactions.^42^ Given that uranium was found to be very efficient in catalyzing initial fullerene formation,^41^ we presumed that if the bottom-up formation mechanism is the main pathway to form fullerenes and endofullerenes, U_2_@C_2*n*_ species must be formed from the very beginning. The formation of U_2_@C_2*n*_ species was first reported in the initial uranium fullerene studies of Smalley and co-workers.^38^ Due to the much higher resolution of the present mass spectrometer used in our studies,^39−42^ in combination with our advances in abilities for mechanistic studies, we decided to reexplore the formation of diuranium EMFs by laser vaporization of graphite-based starting materials doped with uranium metal oxides. The main objective is to unambiguously determine the smallest carbon cage that can encapsulate two U atoms. Figure 1a shows the mass spectrum of fullerenes generated from the laser ablation of graphite doped with 10% of uranium, with the assignment of some key peaks. Significantly, mono- and diuranium encapsulated fullerenes are readily obtained under the present U-doping conditions. These findings are in stark contrast to the results obtained when doping is 1% U, where only mono-uranofullerenes were found.^38^ Besides, the peak of U@C_28_, which was the most abundant at low U-doping, also appears now, but at much lower intensity. The presence of this peak further supports the Carbon Network Growth (CNG) mechanism in mono-uranofullerenes, where the smallest endofullerene U@C_28_ is formed first and subsequent C_2_ incorporation into the closed cage results in cage growth while there is enough carbon vapor to react with the fullerene.^40^ Interestingly, large cages up to U@C_80_ are detected at the present experimental conditions (see Figure S1). Figure 1b shows the relative abundances of diuranium U_2_C_2*n*_ fullerenes generated from our experiment. The abundance distribution exhibits a typical pattern for endohedral fullerenes,^32^ which shows that U_2_@C_48_ is formed in *extremely* low abundance, and U_2_@C_50_ is the first species formed in significant abundance via a bottom-up growth mechanism. The most abundant product is U_2_@C_56_ and cages up to U_2_@C_74_ are detected. It is worth mentioning that in arc discharge methods, the size of endofullerenes is always larger than in laser experiments, probably because of the higher carbon density of the plasma, although other factors such as temperature and inert gas concentrations can also play a role.

Finally, we would like to comment on the peculiar, and rather intense, peak found in the mass spectrum of Figure 1a at around 800 *m*/*z*, with two uranium and an odd number of carbon atoms, U_2_C_27_. Due to the limited size of the C_28_ cage, only one U atom fits inside of it.^42,43^ Therefore, this bizarre species, which remains out of the scope of the present article, could be speculated to be U@C_27_U, similar to the recently detected U@C_27_B.^43^

To identify the limits of fullerene structures to enclose two U atoms inside, we have first studied by DFT methods the smallest stable U_2_@C_2*n*_ EMFs.

### Smallest Endohedral Diuranium Fullerenes

In a systematic theoretical study of Ti@C_2*n*_ fullerenes, it was shown that almost all of the optimal isomers from C_26_ to C_50_ are linked by a simple C_2_ insertion, which provides a strong support for the CNG mechanism.^44^ The experimental data in Figure 1b show that the smallest endofullerene exhibiting a non-negligible abundance is the fullerene with 50 carbon atoms, for which there are 271 possible isomeric cages containing only pentagons and hexagons.^45^

An initial search was performed using the semiempirical AM1 method, to analyze the feasibility of small pristine cages to encapsulate two U atoms. For a cage with 34 C atoms, none of the six isomers has enough space to host the dimer, assuming that the minimum C-C distance to do so is around 7.2 Å. With 40 C atoms, seven isomers would have enough inner space to host the dimer. Nevertheless, the relative energy of these structures is >50 kcal·mol^–1^ (see Figure S3). For C_48_, some isomers are able to host the dimer with an adequately low relative energy (see Figure S4).

**Figure 2:**
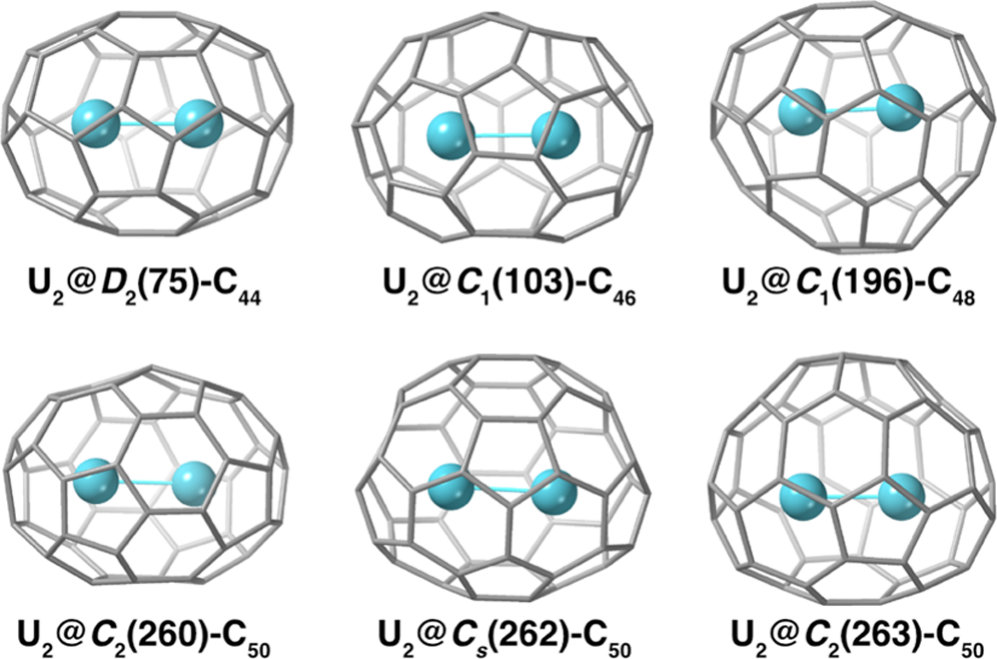
Molecular representations for several U_2_@C_2*n*_ fullerenes (2*n* = 44, 46, 48, and 50) obtained at the DFT PBE level. See Table 1 for selected bond lengths and Figures S3 and S4 for more details on cage selection.

After this screening, the best endohedral candidates were selected and computed at a GGA PBE level. For U_2_@C_50_, *C*_s_(262)-C_50_ was found to be the optimal cage to host two U atoms (Table 1), followed by cages *C*_2_(260)-C_50_ and *C*_2_(263)-C_50_. For U_2_@C_48_, U_2_@C_46_, and U_2_@C_44_, we identified cages *D*_2_(75)-C_44_, *C*_1_(103)-C_46_, and *C*_1_(196)-C_48_ as the optimal fullerenes to encapsulate U_2_ (see Figure 2 and Table S1).

**Table 1:** Relative Energies and Selected Bond Lengths for Several Diuranium Endohedral Fullerenes^a^

cage^b^	2S + 1^c^	Δ*E*	d_U–U_	d_C–U_^d^
*C*_2_(263)-C_50_	1	10.8	2.503	2.39
	3	10.8	2.491	2.40
	5	19.9	2.525	2.39
*C*_s_(262)-C_50_	1	3.8	2.644	2.40
	3	0.0	2.592	2.42
	5	17.5	2.694	2.40
*C*_2_(260)-C_50_	1	5.2	2.654	2.39
	3	2.8	2.622	2.41
	5	13.3	2.688	2.39
*C*_1_(196)-C_48_	1	0.0	2.467	2.36
	3	1.9	2.448	2.37
	5	12.3	2.424	2.37
*C*_1_(103)-C_46_	1^e^	0.0	2.580	2.38
	3	0.1	2.542	2.39
	5	15.5	2.576	2.38
*D*_2_(75)-C_44_	1	0.0	2.501	2.33
	3	0.4	2.448	2.35
	5	22.9	2.464	2.34

a Energies and distances computed at the PBE/TZP level. Further details in Table S2.

b Cage isomers.

c Spin multiplicity.

d Average carbon-uranium distances.

e Open-shell singlet.

**Figure 3:**
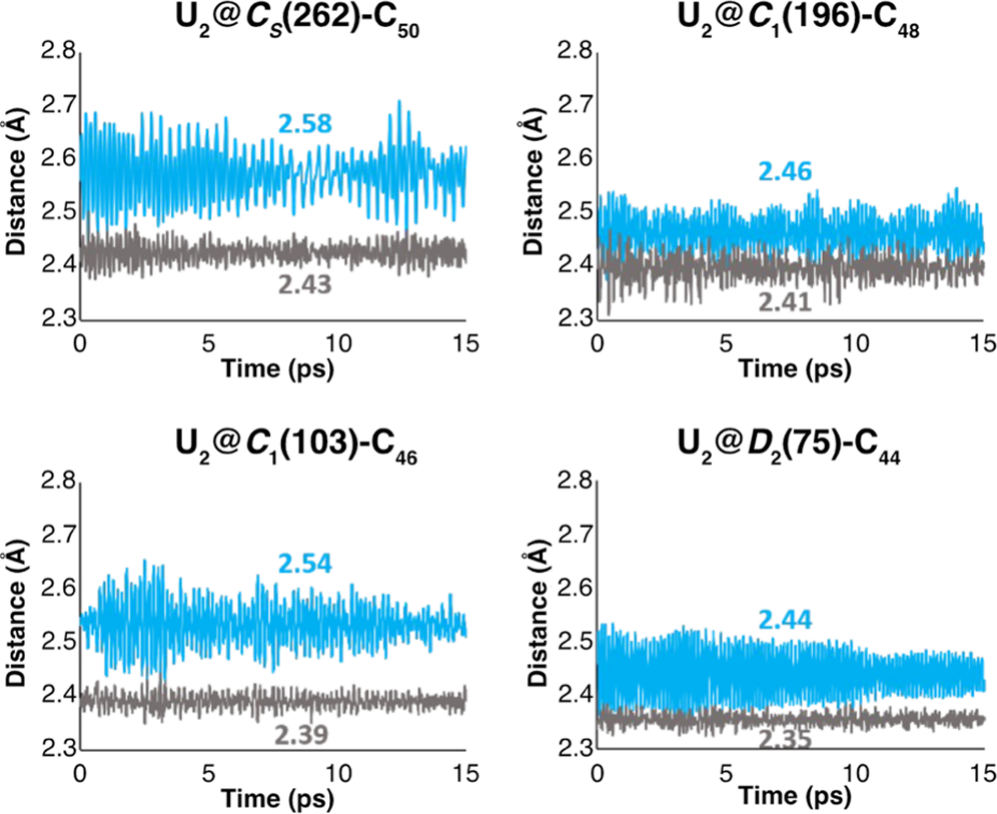
Plot of the U–U (in blue) and C–U distances (in gray) extracted from the CPMD simulations during 15 ps. Average U–U and U–C distances are given in Å. See Table S1 for more details.

To confirm that the C_48_ and C_50_ fullerenes are the smallest cages that can encapsulate the diuranium molecule, we have performed different CPMD simulations at 25 °C using the PBE functional (Figure 3). It is very illustrative to see the significant oscillation of the U–U bond within the *C*_s_(262)-C_50_ cage, around the average value of 2.58 Å. whereas the U–C bond oscillation has a smaller amplitude around 2.43 Å. This means that energy change of the system depends more on the U–C than U–U distance. As the fullerene gets smaller, the C–U distances are forced to contract drastically, decreasing the encapsulation energies and therefore the stability of the endofullerene. Below C_48_, there are several C–U distances shorter than 2.40 Å, which makes the system very reactive and unstable, being its stabilization impossible.

Finally, we recomputed the singlet and triplet states for *C*_2_(260)-C_50_, *C*_s_(262)-C_50_, and *C*_2_(263)-C_50_ using the PBE0 functional. As expected, the metal–metal interaction is enhanced with the hybrid functional, and for all of the electronic states we have observed that the U–U distance is shortened thanks to the presence of the direct interaction between the two actinides (Table S2). The metal–metal bond is analyzed in detail for the U_2_@*I*_h_-C_60_, U_2_@*D*_3h_-C_78_, and U_2_@*I*_h_-C_80_ endofullerenes.

### U–U Bond inside C_60_

**Figure 4:**
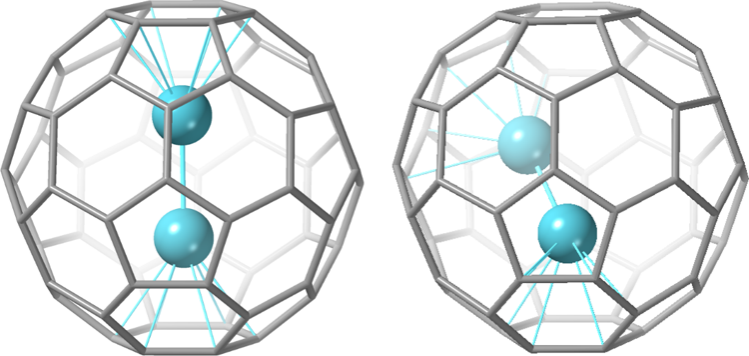
Computed PBE0 structures for U_2_@C_60_. In orientation O1 (left), U_2_ is colinear with one the C_3_ axes of the fullerene, whereas for O2 (right), one of the two U atoms is tilted from the C_3_ axis.

The ability to form metal–metal bonds should hold uranium atoms together during fullerene formation. Although the iconic IPR C_60_(*I*_h_) is not necessarily the carbon cage with the lowest in energy EMF with two uranium atoms inside, this cage is a good model to understand the U–U bond inside an IPR fullerene with a relatively small cavity. MD simulations show that the average U–U distance at room temperature within C_60_ is ∼2.50 Å, which is short enough to generate strong interactions between the two uranium atoms (Figure S5). Two dispositions of U_2_ were found at a static DFT PBE0 level. In orientation O1, U_2_ is colinear with one of the C_3_ axes of the fullerene, while for orientation O2, one of the two U atoms is slightly offset from the most symmetric orientation O1 (see Figure 4). In a naked U_2_ molecule, there are 12e in the valence shell, but in the EMFs, there is a formal transfer of 6e from U_2_ to the fullerene; thus, the U atoms act as U^3+^(f^3^) ions.^28^ Seven bonding and antibonding MOs of σ, π, and ϕ (2) type can be formed with the seven 5f orbitals. Given that U_2_ retains six valence electrons, we have studied the four spin states corresponding to the different spin multiplicities associated with the six electrons. The offset arrangement of U_2_ allows a strong U–U interaction and simultaneous metal–fullerene interactions.

The lowest-energy structure corresponds to the singlet with the σ^2^ π^4^ configuration in which the U–U separation is as short as 2.359 Å. The closed shell configuration is almost degenerate with the σ^2^ π^3^ ϕ^1^ configuration, since it is only 1.0 kcal mol^–1^ above the ground state. The change in the structure is very small since the U–U elongation is only 0.016 Å and the changes in the shortest U–C contacts are also very small (Table 2). In the highest spin quintet and septet states, the relative energies are higher, reaching values of 10.2 and 17.3 kcal mol^–1^, respectively. In the O1 orientation, each U interacts with two distal hexagons and the metal–metal interaction is retained. However, the U–C bond lengths for O1 are longer than for O2, consequently the energies are higher than in O2. The equilibrium between U–U and U–C interactions leads to the quintuplet with configuration σ^1^π^3^ϕ^2^ to be the most stable state for the O1 orientation with only 5.0 kcal mol^–1^ with respect to the singlet O2.

**Table 2:** Relative Energies and Structural and Electronic Parameters for U_2_@C_60_^a^

U2	2S + 1^b^	Δ*E*	d_U–U_	d_U–C_^c^	spin (U_2_)^d^
O1	1	8.7	2.388	2.54	0.000
	3	14.6	2.422	2.51	2.122
	5	5.0	2.453	2.51	4.285
	7	24.3	2.442	2.46	4.498
O2	1	0.0	2.359	2.44	0.000
	3	1.0	2.374	2.46	2.142
	5	10.2	2.394	2.47	4.182
	7	17.3	2.544	2.45	5.850

a Energies in kcal mol^–1^ and bond lengths in Å computed at the PBE0/TZP level. O1 and O2 refer to the two U_2_ orientations observed inside the cavity (Figure 4).

b Spin multiplicity.

c Average U–C bond lengths.

d Spin density on U_2_ unit.

**Figure 5:**
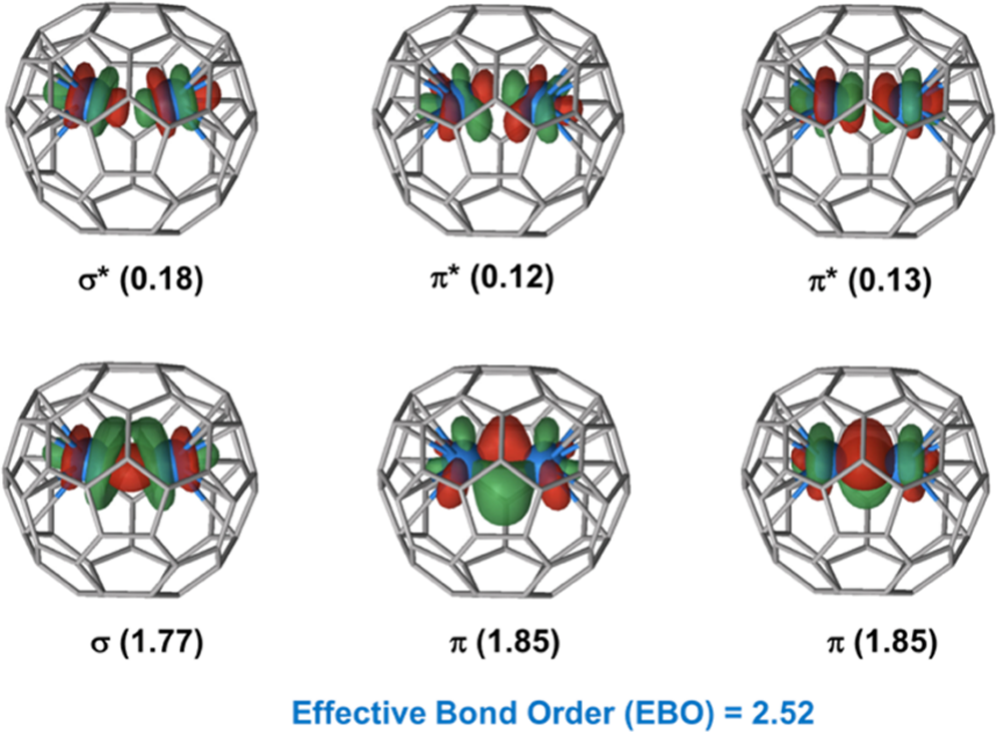
Molecular orbitals contributing to a U–U triple bond for U_2_@C_60_ (orientation O2). Orbital electron populations obtained at the CASSCF level are in parentheses, which give an effective bond order larger than 2.

To verify the electronic structure computed at the DFT level, we performed complete active space second-order perturbation theory calculations (CASPT2). This approach accounts rigorously for the multiconfigurational character of the electronic structure arising from the (possible) presence of multiple unpaired electrons. The CASPT2 calculations carried out for orientation O2 (tilted) confirmed the presence of covalent interactions. At the CASSCF and CASPT2 levels, the ground state is a singlet. Three 5f atomic orbitals per U atom contribute to the actinide-actinide bond. As shown in Figure 5, the covalent bonding in the singlet state is essentially formed by one sigma and two pi orbitals, in which the formal triple bond has an effective bond order (EBO) of 2.52. In the triplet state, with energy very similar to that of the singlet (Table 2), the EBO was also estimated to be equal to 2.52, even though the involved orbitals are slightly different (electronic configuration σ^1^π*_x_*^2^π*_y_*^2^ϕ^1^). See Figures S6 and S7 for more details. Wu and Lu already predicted from DFT calculations that the U_2_ unit should form a bond inside C_60_, although they proposed a ferromagnetic-type interaction.^46^ Similarly, Gagliardi and co-workers also concluded from DFT calculations that the U–U bond inside C_60_ must be considered an artifact due to the constraining size of the cage.^47^ Nevertheless, CASPT2 sets the quintet and septet open-shell states 30 kcal mol^–1^ higher in energy than the singlet dominated by the closed shell configuration. Multireference calculations clearly indicate that the ground state for U_2_@C_60_(*I*_h_) must be interpreted as containing a strong U–U bond formed between 5f orbitals, and the interaction is not magnetic. It is worth mentioning that in the naked U_2_, the quintuple bond is mainly formed by 7s6d orbitals,^13^ whereas in the U^3+^ ions, the 5f orbitals are the main contributors to the covalent bond. This point is extremely relevant since, whereas 7s6d orbitals are fundamental to establish interactions at long distances in some environments or electronic configurations,^48^ the 5f orbitals can play a role in the formation of diuranium endohedrals at short U–U distances.

### U–U Interaction inside U_2_@*D*_3h_(5)-C_78_, U_2_@*I*_h_(7)-C_80_, and U_2_@*D*_2d_(802)-C_104_

**Figure 6:**
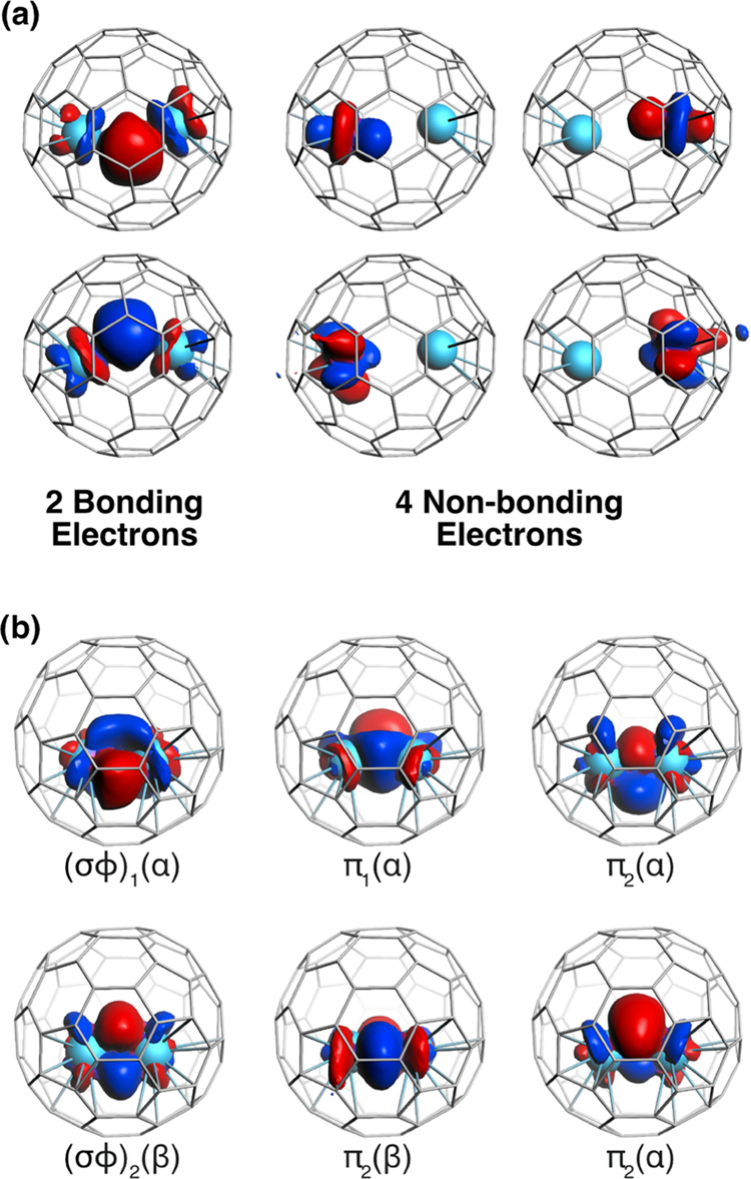
Selected MOs for U_2_@*I*_h_(7)-C_80_ with long and short U–U distances computed at the PBE0 level. (a) Representation of the localized Pipek–Mezey MOs for the septet state with d_U···U_ = 3.793 Å. There are two singly occupied MOs with metal–metal bonding character; the other four MOs with 5f contributions are mainly of a nonbonding character. (b) Representation of the six singly occupied Pipek–Mezey MOs for the triplet state (4α and 2β) with d_U···U_ = 2.406 Å. The six bonding electrons form a triple bond with a formal (σϕ)_1_^1^ (σϕ)_2_^1^(π_1_)^2^(π_2_)^2^ configuration.

Given the presence of strong U–U covalent bonds within C_60_, we have analyzed what happens when the fullerene has a larger cavity. Due to the importance of the *I*_h_(7)-C_80_ cage in the stabilization of EMFs and cluster fullerenes,^22^ we have performed a more detailed analysis for this fullerene, while for *D*_3h_(5)-C_78_ and for *D*_2d_(802)-C_104_, we have only evaluated some electronic states. As for C_60_, two different locations were characterized for U_2_ within the *I*_h_(7)-C_80_ cage. One is dominated by the interaction of the metal with the carbon cage, the two U atoms occupying distal positions with distances longer than 3.7 Å. The second orientation corresponds to a U_2_ forming a strong U–U bond, with bond lengths ranging between 2.37 and 2.65 Å. At the PBE0 level, the lowest-energy structure corresponds to a septet with a U···U separation of 3.793 Å, a value that fits very well with the experimental value of 3.842 Å (see Figure S8). This structure is clearly dominated by optimization of the U–fullerene interaction; however, a U–U interaction remains, as shown by the localized Pipek–Mezey MOs represented in Figure 6a.

An estimate of the bond energy was obtained by calculating the energy of the broken symmetry (BS) singlet state, which is found at 8.9 kcal mol^–1^ above the septet state (Table 3). According to Mulliken population, the two U atoms have an atomic spin density +3.10 e, while in the BS state, the populations are +2.45 and −2.45 e. The presence of direct metal–metal interaction is evidenced by the increase of the U···U separation from 3.794 Å in the septet to 3.889 Å when the BS singlet is relaxed. The energy of the BS state decreases 1.6 kcal mol^–1^ when geometry relaxation is allowed. It should be noted that in the U_2_@*D*_2_-C_104_ endofullerene, the U···U distance reaches the value of 6.250 Å, and at this separation, the singlet BS and the septet state are degenerate (Table 3). This cage was selected because it corresponds to the *I*_h_(7)-C_80_ carbon cage, with 24 extra atoms added between the two hemispheres of the fullerene (Figure S9). Similar calculations were performed for U_2_@*D*_3d_(5)-C_78_, where the two metal atoms are 4.240 Å apart. At this distance, the septet is still below the BS singlet by 4.5 kcal mol^–1^.

**Table 3:** Structural and Electronic Parameters for Several Diuranium Endohedral Fullerenes^a^

compound	bond	2S + 1^b^	Δ*E*^c^	d_U–U_^d^	spin^e^
U_2_@*I*_h_-C_80_	long	1	26.1	3.846	
		3	17.6	3.917	1.07/1.07
		5	10.0	3.847	2.09/2.09
		7	0.0	3.794	3.10/ 3.09
		1^BS^	8.9	3.794	2.45/–2.45
	short	1	9.4	2.370	
		3	7.4	2.406	1.06/1.06
		5	14.5	2.519	3.29/0.76
		7	19.8	2.642	3.10/3.09
U_2_@*D*_3h_(5)-C_78_	long	7	0.0	4.240	2.64/ 3.06
		1^BS^	4.5	4.240	2.57/–2.15
	short	1	14.7	2.396	
		3	10.0	2.436	0.63/1.42
U_2_@*D*_2d_(802)-C_104_	long	7	0.0	6.250	2.95/2.95
		1^BS^	0.0	6.250	2.95/–2.95

a All values were determined at the PBE0/TZP level.

b Spin multiplicity. 1^BS^ represents broken symmetry state computed at the geometry of septet state.

c Relative energies in kcal mol^–1^.

d Bond lengths in Å.

e Mulliken spin densities.

Endo-fullerenes with short U–U bonds were also characterized as minima with moderate energies with respect to the structures with large metal–metal separations. Hence, a triplet state with a (σϕ)_1_^1^(σϕ)_2_^1^(π_1_)^2^(π_2_)^2^ electronic configuration and a U–U bond length of 2.406 Å was found only 7.4 kcal·mol^–1^ above the septet with a d_U···U_ = 3.793 Å. The singlet with a σ^2^π^4^ configuration and a d_U···U_ somewhat shorter (2.370 Å) is 2 kcal·mol^–1^ above the triplet.

**Figure 7:**
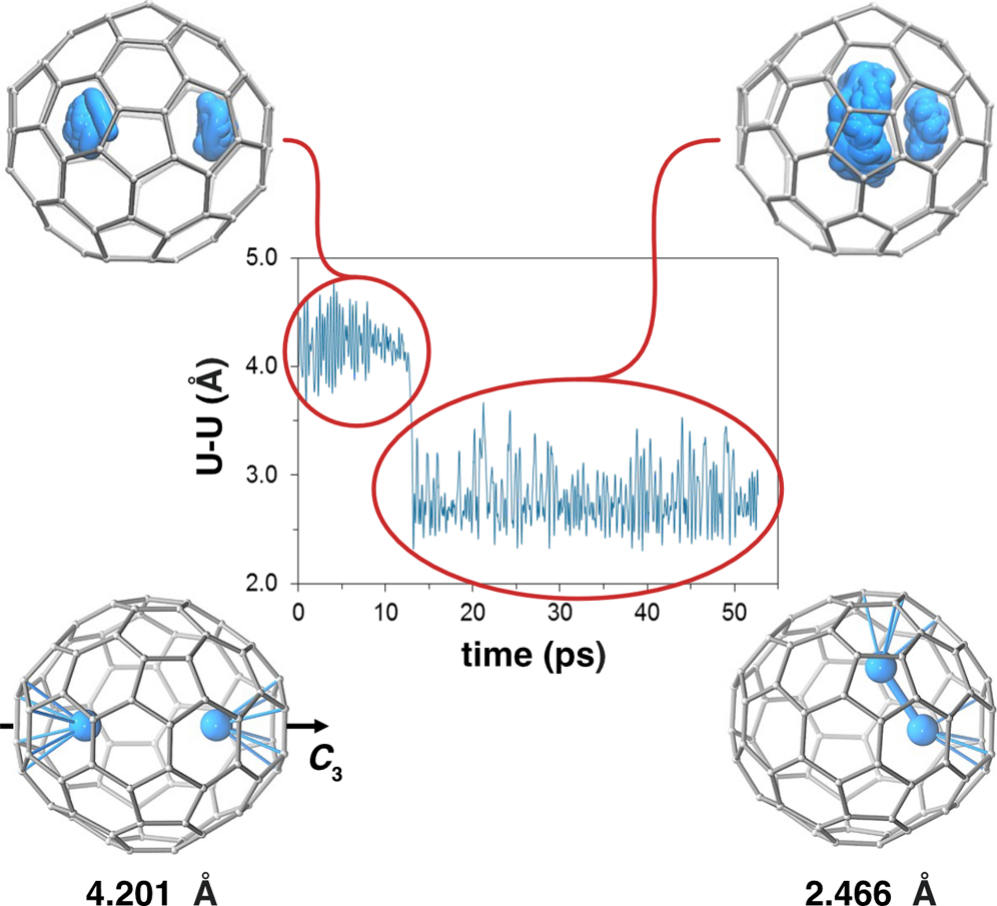
Car–Parrinello Molecular Dynamics simulations (top) and DFT-optimized structures with U–U distances for U_2_@C_78_. Representation of the motion of the U atoms inside U_2_@*D*_3h_(5)-C_78_ for a 50 ps MD simulation time. Only U–C contacts smaller than 2.6 Å are represented in the optimized structures.

The really short U–U bond lengths together with MOs represented in Figure 6b support the idea that thanks to the overlap, mainly between f orbitals, a strong triple bond is formed between two U^3+^ ions. Indeed, for U_2_@*D*_3h_(5)-C_78_, we have observed the formation of direct U–U bonds during CPMD simulations. We started the simulation with the U ions along the C_3_ axis with a metal–metal separation of ∼4 Å. Initially, the two ions remained near the initial positions; however, after 13 ps, one U escaped to the region defined by the σ_h_ plane perpendicular to the C_3_ axis. As shown in Figure 7, this U atom moves around the C_3_ axis during the rest of the simulation (∼50 ps) at about 2.7 Å from the other U. The sustained short distance is a clear indication that a strong U–U bond can exist between the two U ions in addition to the initial situation with two separated (nonbonded or weakly bonded) U atoms and it is likely that the metal–metal interactions are important during the process of endohedral fullerene formation.

We have also analyzed the influence of the spin–orbit coupling in U_2_@*I*_h_(7)-C_80_ when d_U···U_ = 2.406 Å (short distance, see Computational Method for details). The presence of six spinors (Figure S10) with densities that resemble considerably the MO isosurfaces represented in Figure 6b leads us to think that the description of a strong triple U–U bond is qualitatively correct. Energetically, however, this short disposition becomes somewhat more destabilized compared to the orientation with a long U–U distance (now around 20 kcal mol^–1^), which indicates that the triple bond is likely weakened, in line with the results recently obtained for the naked U_2_ dimer.^20^

Finally, CASSCF/CASPT2 calculations for the short U–U distance within *I*_h_-C_80_ were performed. Overall, the results are very consistent with those found for U_2_@C_60_; the ground state is a singlet with a σ^2^π*_x_*^2^π*_y_*^2^ configuration, in which the formal triple U–U bond has an EBO = 2.57. CASSCF MOs (Figure S11) are rather similar to those obtained at the DFT level in Figure 6b. For the long U_2_ arrangement, different electronic configurations were found: One with the six valence electrons of the two U atoms located on the 5f atomic orbitals that show no effective bonding between the two metal atoms (Figure S12); states with other configurations involving also 7s and 6d atomic orbitals have relative energies that are within a few kcal mol^–1^ below or above the configuration with the six electrons in the 5f orbitals. In one of them, an EBO = 0.55 was found (Figures S13, S14). Table S4 summarizes the computed CASSCF and CASPT2 energies, which clearly show that for the short bond length, the ground state is a singlet, whereas for the long separation, the energy difference between the singlet and septet is less than 2 kcal·mol^–1^. The comparison between CASPT2 and PBE0 must be done with caution due to the multiconfiguration character of the electronic structure. Moreover, at the CAS level, we are not able to include cage orbitals in the active space. See the SI for more details.

Considering the DFT and CASSCF results we can conclude that, at U–U separations close to 4 Å, an effective interaction can exist thanks to the non-negligible contribution of 7s6d orbitals to the bonding MOs (Figure 6a). As shown by atomic orbital contributions in Table S3, less than 2e would be involved in the U–U interaction, with a bond energy lower than 10 kcal mol^–1^. Previous studies using a GGA functional suggested that two effective electrons are involved in the formation of a bonding interaction between the two uranium atoms within *I*_h_(7)-C_80_;^28^ however, the authors proposed that the interaction is stronger and related to 5f orbitals. All this contrasts with the Th_2_@*I*_h_(7)-C_80_ system, where the two valence electrons of the Th^3+^ ions form a strong covalent bond (> 40 kcal mol^–1^ at 3.816 Å),^34^ which could be partially due to the smaller contribution of 5f orbitals to the sigma bonding molecular orbital in the case of the Th endofullerene (Table S3). The overlap between the 5f orbitals does not contribute significantly to the An–An bonding at distances close to 4 Å (Figure S15).

## Conclusions

Orbitals 7s and 6d of U have a predominant role in the formation of a multiple metal–metal bond for the naked U_2_ molecule. Encapsulated within a fullerene there is a formal transfer of six electrons from U_2_ to the fullerene orbitals, thus the U^3+^ ions have 5f^3^ electronic configurations. DFT and CASSCF/CASPT2 calculations in combination with CPMD simulations reveal that both U_2_ arrangements, with short and long U–U separations, are present in larger fullerenes like C_78_ and C_80_, whereas confined within C_60_, the U ions exhibit metal–metal bond lengths smaller than 2.5 Å with effective bond orders close to 2.5. The difficulty in characterizing this bond by X-ray diffraction arises from the competition between the formation of the triple U–U bond and the interaction of the two U atoms with the cage. Hence, the U_2_ arrangement with d_U–U_ = 2.406 Å in U_2_@*I*_h_(7)-C_80_ is computed to be about 7 kcal·mol^–1^ above the lowest-energy structure with the two U atoms at 3.793 Å, which matches well the experimental one of 3.842 Å. At this distance, two electrons were found located in two singly occupied bonding U–U molecular orbitals, still providing some covalent interaction between the two metal centers. Indeed, the energy difference between septet and open-shell singlet is 8.9 kcal mol^–1^. This value decreases to 4.5 kcal mol^–1^ in U_2_@C_78_, where d_U–U_ = 4.240 Å, and is almost zero in U_2_@C_104_, with d_U–U_ = 6.251 Å. Therefore, our results confirm that although the orbital overlap between 5f–5f decreases monotonically with the U–U distance, the presence of a non-negligible U···U interaction takes place at distances even above to 4 Å thanks to the overlap between hybrid 7s6d orbitals. Such interactions were also confirmed at the CASSCF/CASPT2 level.

The gas-phase synthesis of diuranium endofullerenes U_2_@C_2*n*_ with 2*n* ≥ 50, obtained from laser ablation of graphite doped with 10% uranium, reinforces the hypothesis that this kind of systems can only be stabilized thanks to the presence of important metal–metal interactions. We are still far from fully understanding how fullerene formation occurs. However, in the bottom-up approach, the electrons retained by the metal help hold the metals close, which is a necessary requirement to form the dimetallic endofullerenes in the atmosphere. In the absence of M–M interactions, none of the U_2_@C_2*n*_ would ever form since they would have to occur purely statistically, an almost impossible trajectory. Therefore, we should see M_2_ acting more like a template than as two cations that tend to be far apart due to strong repulsions between them. The nature of M–M interactions in the closed fullerene depends on the size of the fullerene cavity.

## Experimental Section

### Gas-Phase Synthesis and Detection of U_2_@C_2*n*_

The starting materials graphite (99.9999%, 2–15 μm) and UO_2_ (>99.9% purity) are thoroughly mixed in a ratio 90:10 and then molded into a composite rod by compression to result a 10 atom % U-doped carbon target rod. U_2_@C_2*n*_ are formed *in situ* by use of a pulsed supersonic cluster source by a single laser pulse of a Nd:YAG laser under a flow of helium (see below).^40,41^ The gas-phase reaction products were analyzed by a custom-built 9.4 T FT-ICR mass spectrometer directly coupled to the cluster source, and the analysis was conducted with positive ions.^49,50^ Evaporation of a translating and rotating target rod (12.7 mm diameter) is achieved by a single laser shot fired from a Nd:YAG (532 nm, 3–5 ns pulse width, ∼1.5 mm beam diameter, 5 mj per pulse) in conjunction with the opening of a pulsed valve (800 ms duration) to admit He flow over the sample. Carbon vapor produced then enters a channel 4 mm in diameter and ∼8.5 mm in length. The laser is fired ∼2 ms after opening of the pulsed valve for evaporation of metal-doped graphite samples. Ions produced by 10 individual vaporization events were accumulated and transferred by octopoles to an open cylindrical trap ICR cell (70 mm diameter, 212 mm long, aspect ratio ∼2). The ions are then accelerated to a detectable radius by a broadband frequency sweep excitation (260 Vp-p, 150 Hz μ^–1^, 3.6 down to 0.071 MHz) and detected as the differential current induced between two opposed electrodes of the ICR cell. Each of the acquisitions is Hanning-apodized and zero-filled once before fast Fourier transform and magnitude calculation. Up to ten time-domain acquisitions are averaged. The experimental event sequence is controlled by a modular ICR data acquisition system.^40,41^ The positively charged molecular ions are expected to be representative of the neutral abundance distribution generated by laser vaporization. However, we note that the corresponding neutrals may exhibit different stabilities.

### Computational methods

DFT calculations for U_2_@C_2*n*_ EMFs were carried out with the ADF 2019 package^51^ using PBE and PBE0 exchange-correlation functionals in combination with Slater TZP basis sets to describe the valence electrons of U and C. Frozen cores were described by means of single Slater functions, consisting of the 1s shell for C and the 1s to 5d shells for U. Scalar relativistic corrections were included by means of the ZORA formalism. Dispersion corrections by Grimme were also included.^52^ Localized molecular orbitals were obtained with Pipek–Mezey method.^53^ For the spin–orbit calculations, we carried out single-point energy calculations (PBE0/TZP) at the geometries optimized at the PBE0/TZP + scalar relativistic level. The noncollinear approximation, in which in each point in space the spin-polarization can have a different direction, was considered.

CASPT2 calculations were performed with OpenMolcas.^54^ Except for the U_2_@C_80_ conformation with the long U–U distance, the geometries of the molecules do not have symmetry elements. The *C*_2h_ symmetry group is the highest Abelian group that could be applied for U_2_(long)@C_80_ system. The active space contains six orbitals and six electrons in the U_2_@C_60_ calculations and for U_2_@C_80_ conformation with the short U–U distance. Test calculations with larger active spaces did not alter the results in these cases; U–carbon bonding and antibonding orbitals enter the active space with occupation numbers close to 2 and 0, respectively. For the U_2_@C_80_ conformation with the longer U–U distance, the active space was extended to 13 orbitals and six electrons to correctly describe all low-lying electronic configurations, see Figures S12 and S13 for the character of these active orbitals. State average calculations were performed for the six lowest states in each irreducible representation and spin moment. Scalar relativistic effects are taken into account with the Douglas–Kroll–Hess Hamiltonian.^55^ We have used the standard IPEA = 0.25 zeroth-order Hamiltonian in the CASPT2 calculations and applied an imaginary level shift of 0.10 E_h_ to avoid intruder states. The basis set is taken from the ANO-RCC internal library^56^ of OpenMolcas using the following contractions U(8s,7p,5d,3f,1g) and C(2s,1p). Doubling the basis set on the carbon case leads to changes that are smaller than 1 kcal mol^–1^ in the relative energies of the U_2_@C_60_ molecules.

Molecular dynamics simulations were carried out using Car–Parrinello Molecular Dynamics (CPMD) program.^57^ The description of the electronic structure was based on the expansion of the valence electronic wave functions into a plane wave basis set, which was limited by an energy cutoff of 100 Ry. The interaction between the valence electrons and the ionic cores was treated through the pseudopotential (PP) approximation (Martins–Troullier type for C and Goedecker–Teter–Hutter type for U).^58−60^ The PBE functional was selected and Grimme dispersion corrections were included. The simulations were carried out in a cubic cell with a side length of 13.5 Å for U_2_@C_80_ and U_2_@C_78_, and 12.5 Å for U_2_@C_60_. All of the systems were simulated using a local spin density approximation with septet multiplicities for U_2_@C_80_ and U_2_@C_78_ and singlet for U_2_@C_60_.

A data set collection of computational results is available in the ioChem-BD repository and can be accessed via http://dx.doi.org/10.19061/iochem-bd-2-59.61.^61^

## Supporting Information Available

Experimental mono-uranium formation distribution; mass scale expansions of the FT-ICR mass spectrum; relative energies and distances for small cages; CPMD results; PBE0 and CASSCF MOs for endohedral uranofullerenes; results from spin–orbit calculations; and CASSCF and CASSPT2 energies (PDF)
