# Supplementary material for: Are U–U Bonds Inside Fullerenes Really Unwilling Bonds?
Source: J Am Chem Soc. 2023 Mar 6;145(12):6710–8. doi: 10.1021/jacs.2c12346 (PMC10064334; doi:10.1021/jacs.2c12346)
Supplement: Supplementary file 1 — ja2c12346_si_001.pdf [file ja2c12346_si_001.pdf]

Supplementary Information

Are U-U bonds inside fullerenes really unwilling bonds?

Antonio Moreno-Vicente,[a] Yannick Roselló,[a] Ning Chen,[b] Luis Echegoyen,[c] Paul W. Dunk,[d] Antonio Rodríguez-Fortea,*,[a] Coen de Graaf *,[a,e] and Josep M. Poblet*,[a]

[a] Departament de Química Física i Inorgànica, Universitat Rovira i Virgili, Marcel·lí Domingo 1, 43007 Tarragona, Spain

[b] Laboratory of Advanced Optoelectronic Materials, College of Chemistry, Chemical Engineering and Materials Science, Soochow University, Suzhou, Jiangsu 215123, PR China

[c] Department of Chemistry, University of Texas at El Paso, 500 West University Avenue, El Paso, Texas 79968, United States

[d] Ion Cyclotron Resonance Program, National High Magnetic Field Laboratory, Florida State University, Tallahassee, FL 32310, USA

[e] ICREA, Pg. Lluis Companys 23, Barcelona 08010, Spain

Table of Contents

Supplementary figures ....Page

Fig. S1. Mono-uranium formation distribution in laser ablation experiments...................................3

Fig. S2. Mass scale expansion of the FT-ICR mass spectrum...4

Fig. S3. Relative energies and distances C_{34} to C_{44}..5

Fig. S4. Relative energies and distances C_{46} to C_{50}..6

Fig. S5. CPMD $U_2@C_{60}$..6

Fig. S6. Canonical MOs $U_2@C_{60}$ (O1)...7

Fig. S7. Canonical MOs $U_2@C_{60}$ (O2)...8

Fig. S8. PBE0 structures for $U_2@C_{80}$...9

Fig. S9. $U_2@D_2$-C_{104}..9

Fig. S10. Representation of six spinors from spin-orbit calculations…………..……………..……10

Fig. S11. $U_2@I_h$-C_{80} CASSCF MOs at 2.406 Å…...10

Fig. S12. $U_2@I_h$-C_{80} CASSCF MOs at 3.794 Å...11

Fig. S13. Natural orbitals of the 3rd singlet CASSCF root of B_{1g} symmetry....................................11

Fig. S14. Natural orbitals of the 3rd triplet CASSCF root of A_{1g} symmetry.....................................12

Fig. S15. U-U atomic orbital overlap..12

Supplementary tables...13

Table S1. Average $d_{U\text{-}U}$ and $d_{C\text{-}U}$ for CPMD simulations..13

Table S2. Relative energies, U-U and C-U distances, spin densities and S^2.....................................13

Table S3. Atomic orbital contributions (in %) at PBE0/TZP level for $U_2@I_h(7)$-C_{80} and................14

Table S4. Relative CASSCF and CASPT2 energies...14

Computed structures……...15

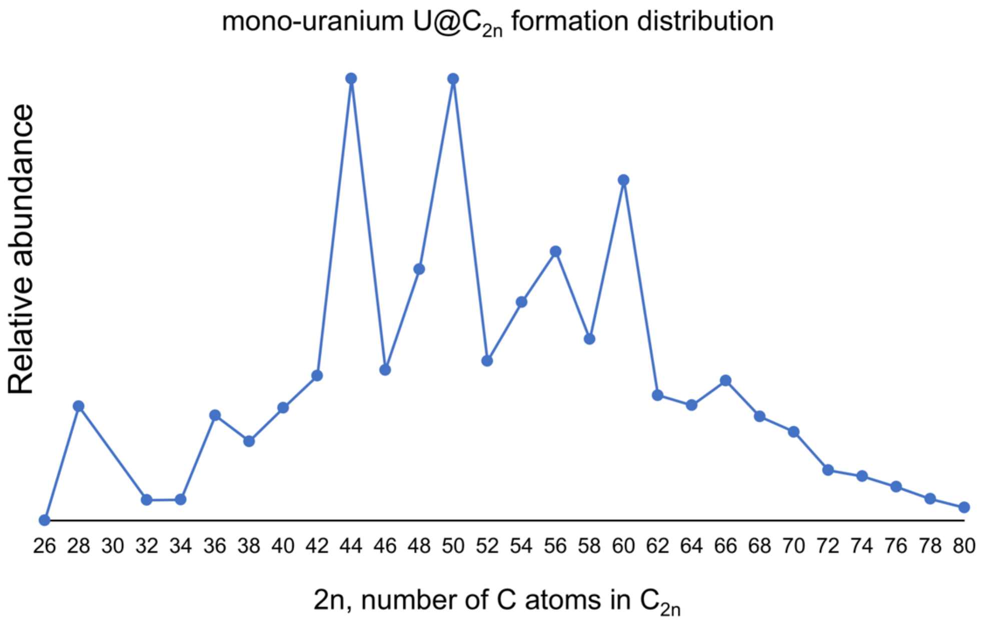

Figure S1. Relative abundances for $U@C_{2n}$ species obtained in gas phase from laser ablation of graphite doped with 10% of uranium.

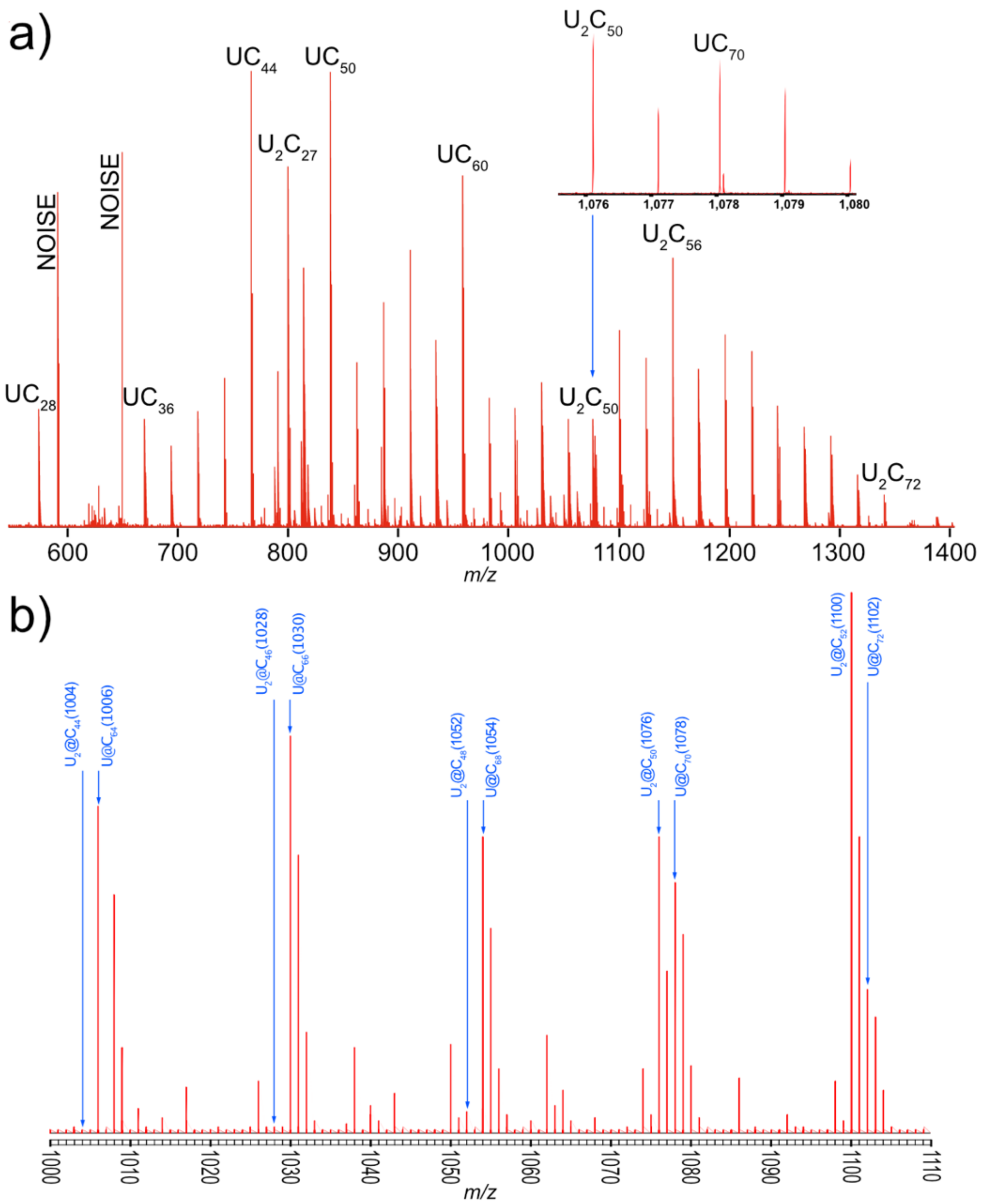

Figure S2. (a) FT-ICR mass spectrum of cluster cations formed from laser ablation of graphite doped with 10% of uranium. Peaks for $U@C_{2n}$ and $U_2@C_{2n-20}$ show only differences of 2 units in the mass spectrum (U: 238 and 20 C: 240) and in a large scale they appear as overlapped. Mass scale expansion from m/z=1076 to 1080 is shown in the inset, where the peaks of $U_2@C_{50}$ and $U@C_{70}$ are clearly separated. (b) Mass scale expansion of the FT-ICR mass spectrum from m/z = 1000 to 1110 and peak assignments for mono- and di-uranium EMFs.

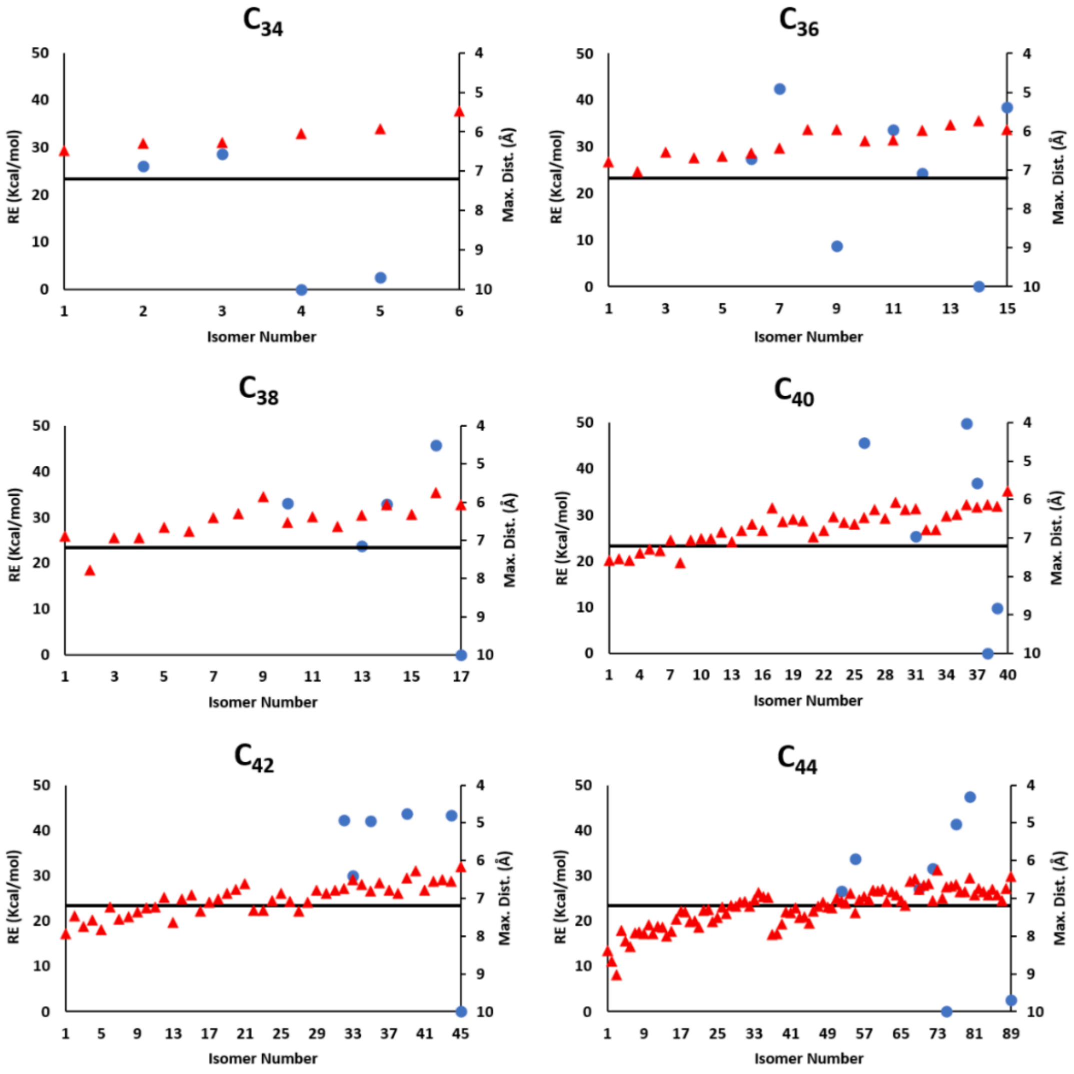

Figure S3. Plot of the relative energies (AM1 level) (left y axis) and maximum carbon-carbon distances (right y axis) for all the isomers from C_{34} to C_{44} fullerene families. Blue dots are the relative energies, red triangles are the maximum distances, and the black line represents a minimum distance of 7.2 Å. To estimate this arbitrary minimum distance, we have considered $d_{min} = 2*d_{U\text{-}C} + d_{U\text{-}U}$, with $d_{U\text{-}C} = 2.4$ Å and $d_{C\text{-}C} = 2.4$ Å. For RE, we have represented only those with values lower than 50 kcal·mol^{-1}.

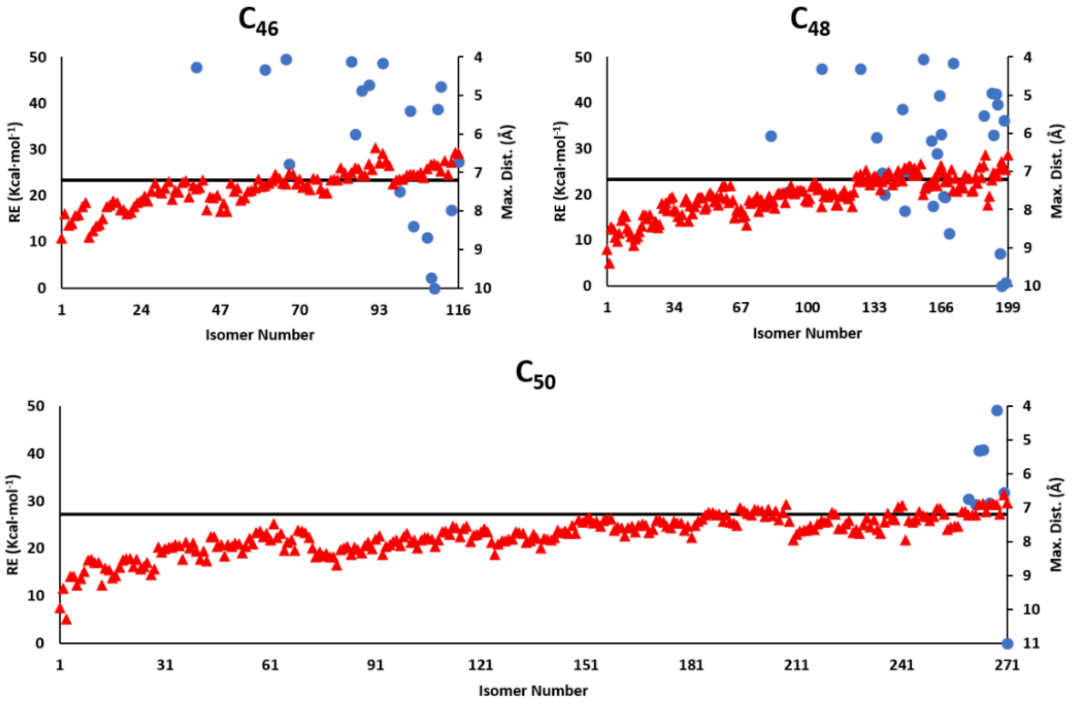

Figure S4. Plot of the relative energies (AM1 level) (left y axis) and maximum carbon-carbon distances (right y axis) for all the isomers from C_{46} to C_{50} fullerene families. Blue dots are the relative energies, red triangles are the maximum distances, and the black line represents a minimum distance of 7.2 Å. To estimate this arbitrary minimum distance, we have considered $d_{min} = 2*d_{U\text{-}C} + d_{U\text{-}U}$, with $d_{U\text{-}C}$ = 2.4 Å and $d_{C\text{-}C}$ = 2.4 Å. For RE, we have represented only those with values lower than 50 kcal·mol^{-1}.

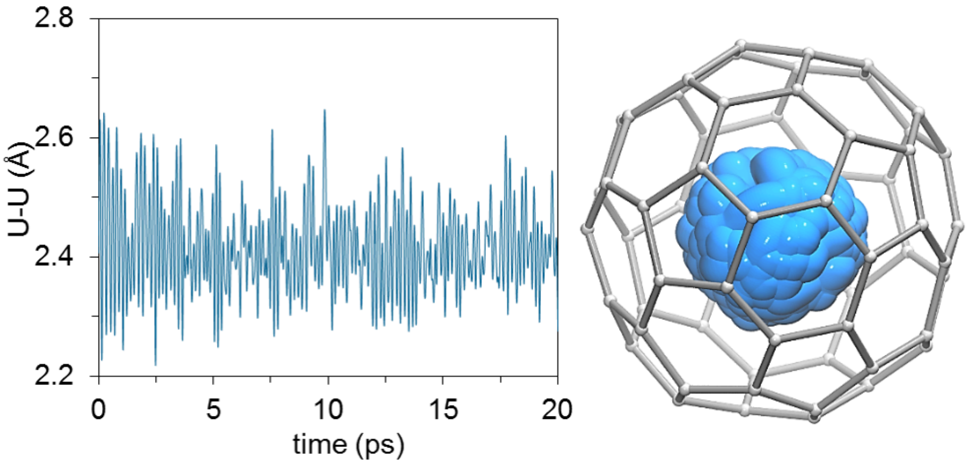

Figure S5. CPMD uranium-uranium distances ($d_{U\text{-}U}$) for $U_2@C_{60}$ during a 20-ps simulation.

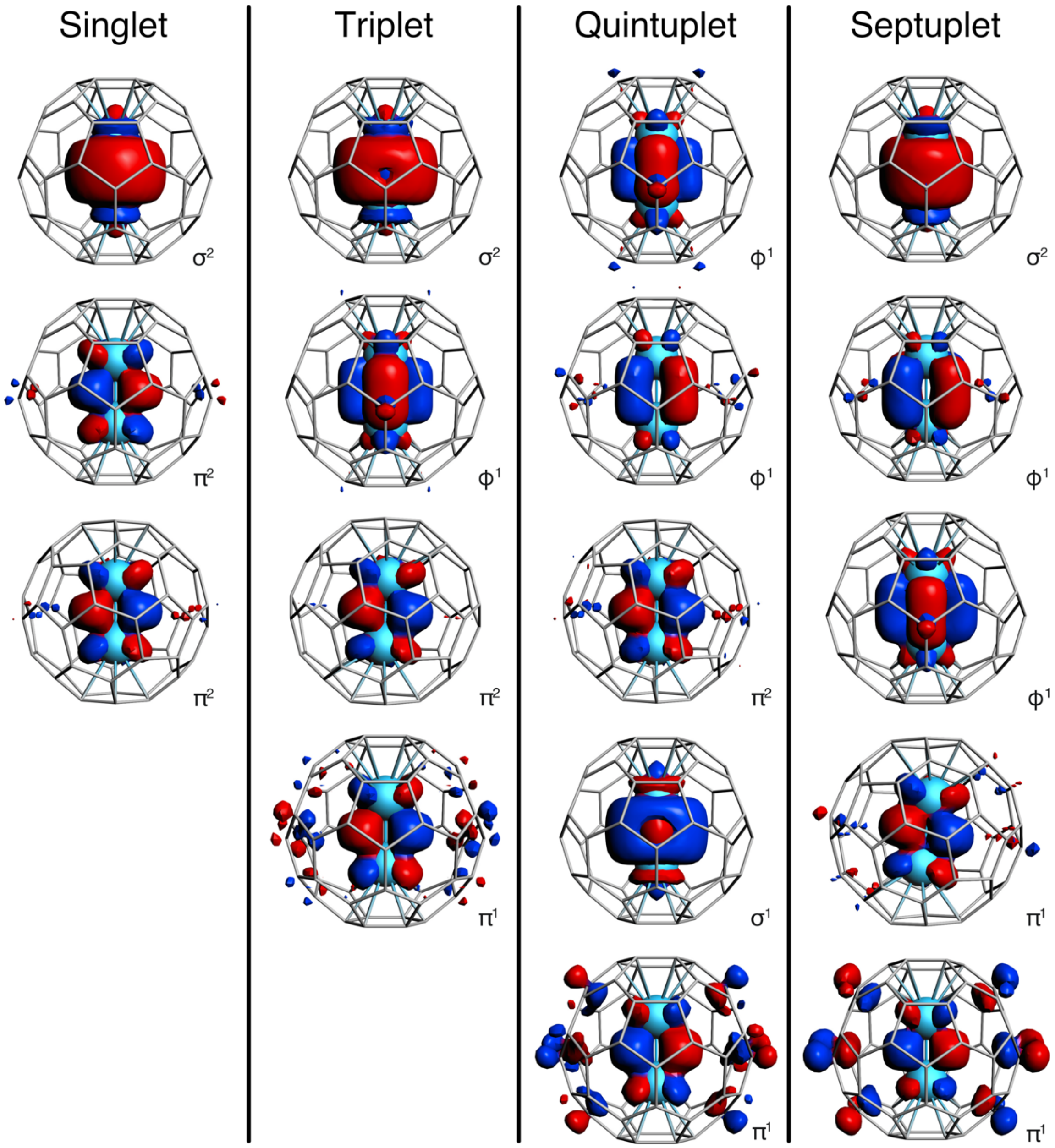

Figure S6. Canonical MOs for O1 in $U_2@C_{60}$. Temptative configurations are $\sigma^2\pi^4$ for singlet, $\sigma^2\pi^3\phi^1$ for triplet, $\sigma^1\pi^3\phi^2$ for quintuplet, $\sigma^2\pi^2\phi^2c^2$ for septuplet, where c^2 refers to electrons located on the carbon cage.

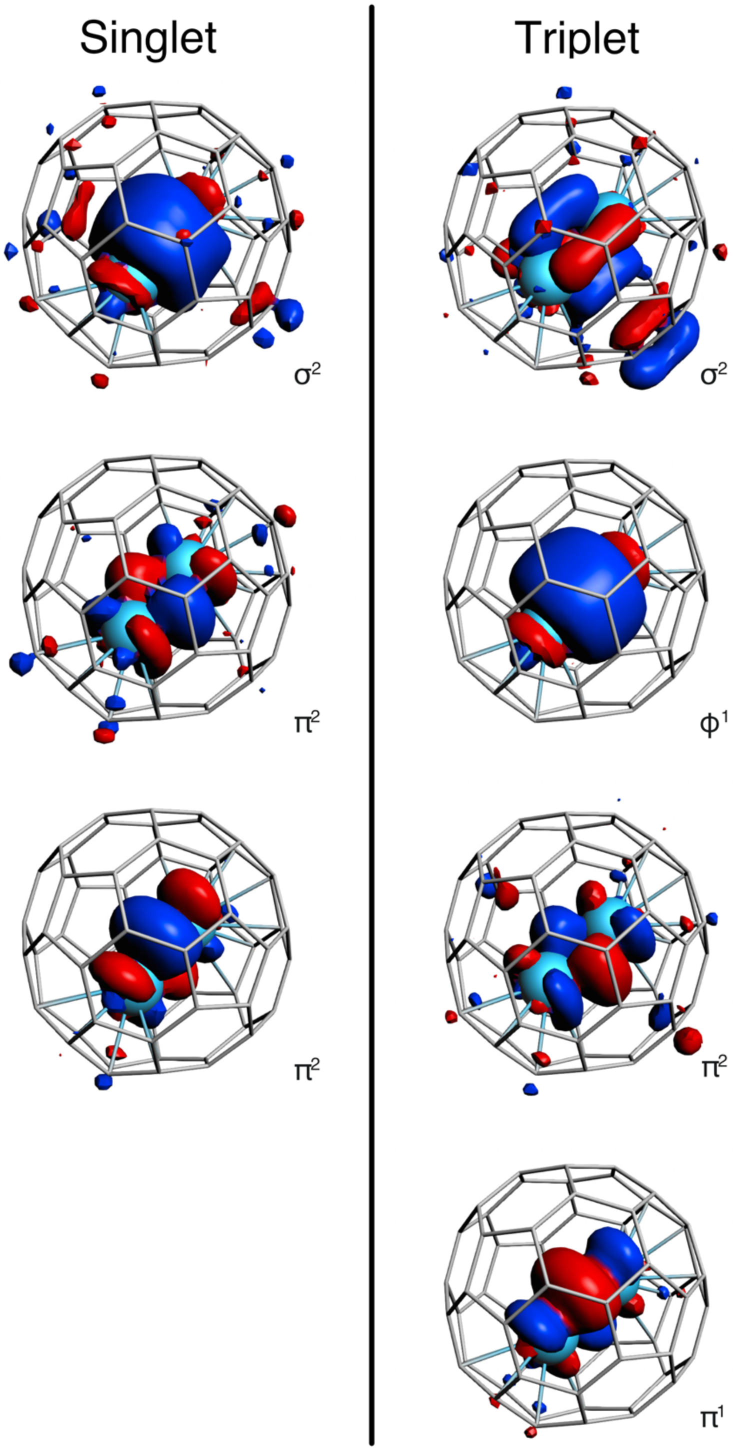

Figure S7. Canonical MOs for O2 in $U_2@C_{60}$. Temptative configurations are $\sigma^2\pi^4$ for singlet, $\sigma^2\pi^3\phi^1$ for triplet.

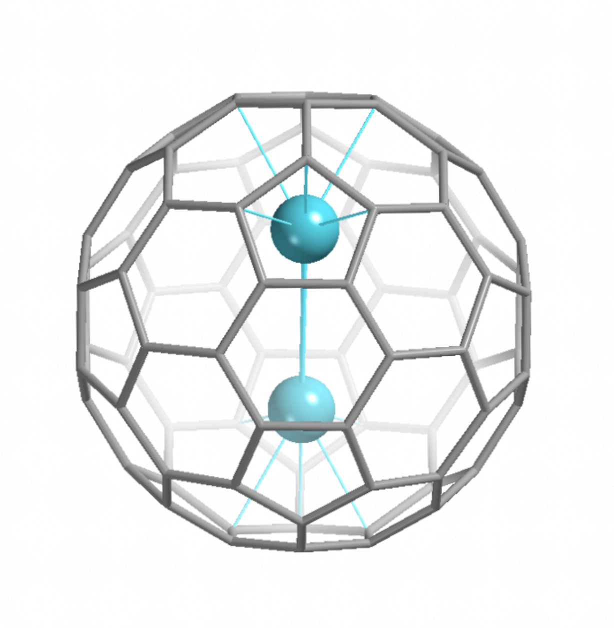

Figure S8. The computed PBE0 structures for $U_2@C_{80}$. The most stable orientation (right) is almost colinear with C_3 axes of the fullerene with a U-U distance of 3.793 Å. Meanwhile, the offset orientation has a stronger U-U bond between 2.37 and 2.65Å.

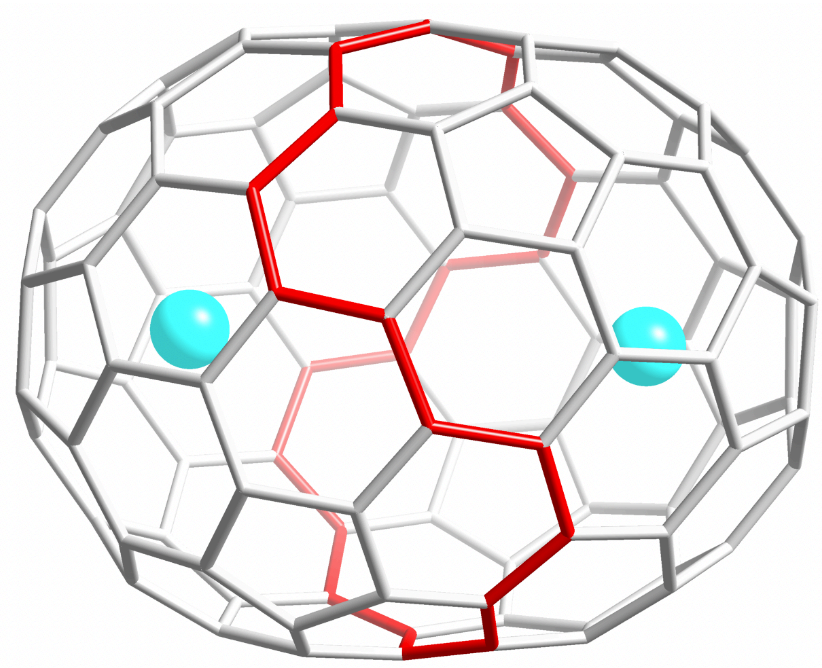

Figure S9. $U_2@D_2$-C_{104}, the U···U distance is 6.25 Å. The 24 carbons highlighted in red are those needed to form D_2-C_{104} from $I_h(7)$-C_{80}.

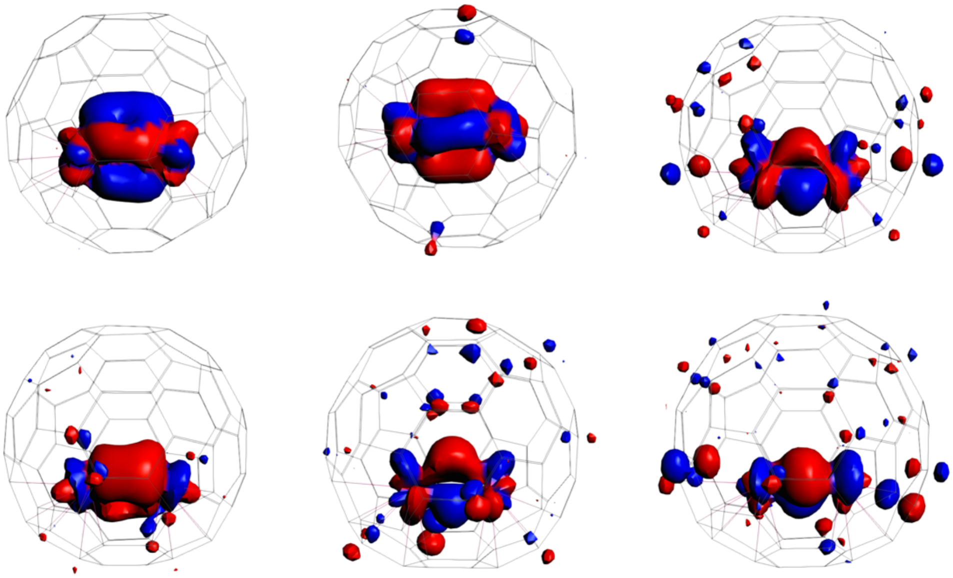

Figure S10. Representation of the density isosurfaces related to the six spinors, obtained from the calculations that include spin-orbit coupling, that resemble MOs in Figure 6b. There is no possibility of localizing more these densities in the U_2 unit with the software used (ADF 2019). The qualitative description of the triple bond remains valid when spin-orbit coupling is considered.

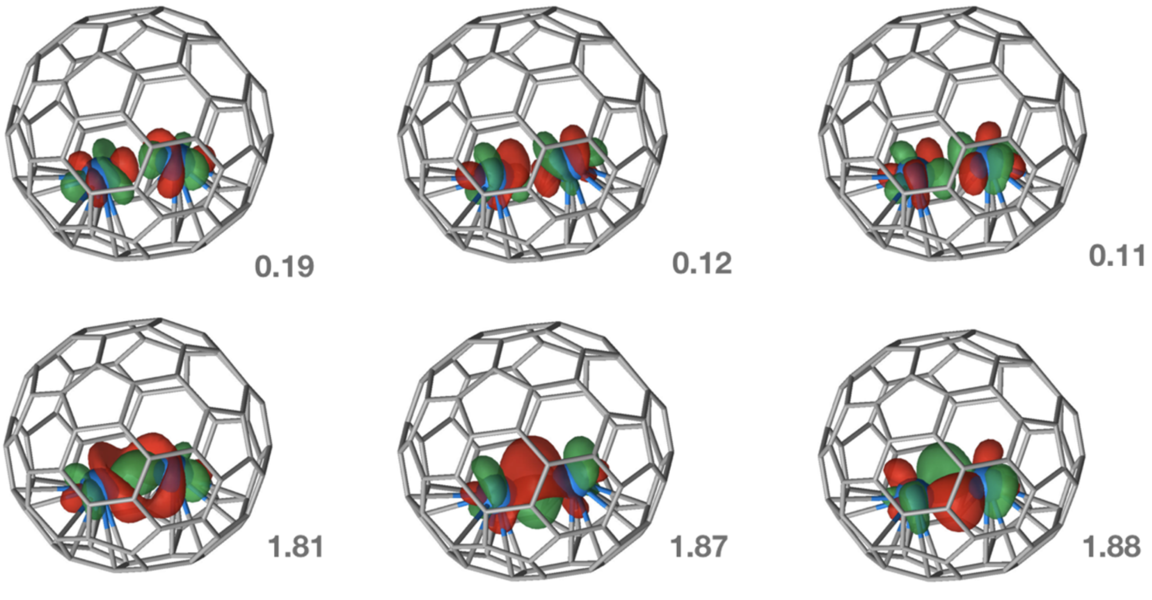

Figure S11. CASSCF molecular orbitals computed for the lowest singlet state of $U_2@I_h$-C_{80} at U-U bond length of 2.406 Å. The MO occupations yield an effective bond order (EBO) of 2.57.

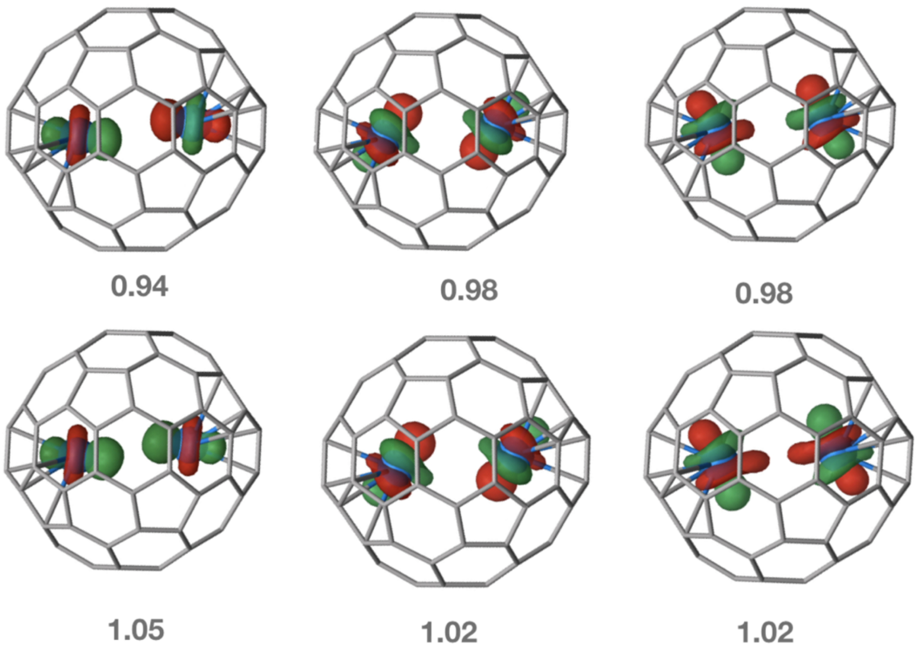

Figure S12. CASSCF molecular orbitals computed for the lowest singlet states of $U_2@I_h$-C_{80} at U-U bond length of 3.794 Å. 5f atomic orbitals show no effective bonding between the two metal atoms.

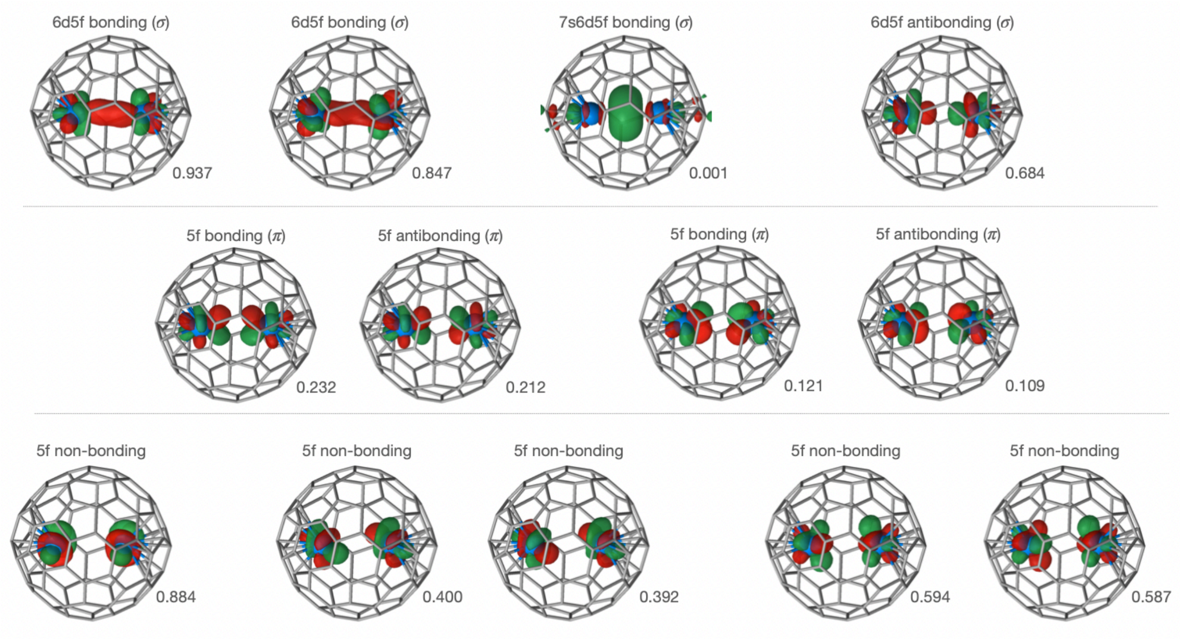

Figure S13. Natural orbitals and natural occupation numbers of the 3rd singlet CASSCF root of B_{1g} symmetry. ΔE(CASSCF) = 5.1 kcal/mol, ΔE(CASPT2) = -0.2 kcal/mol. Estimated BO: (0.937 + 0.847 + 0.001 - 0.684)/2 = 0.55.

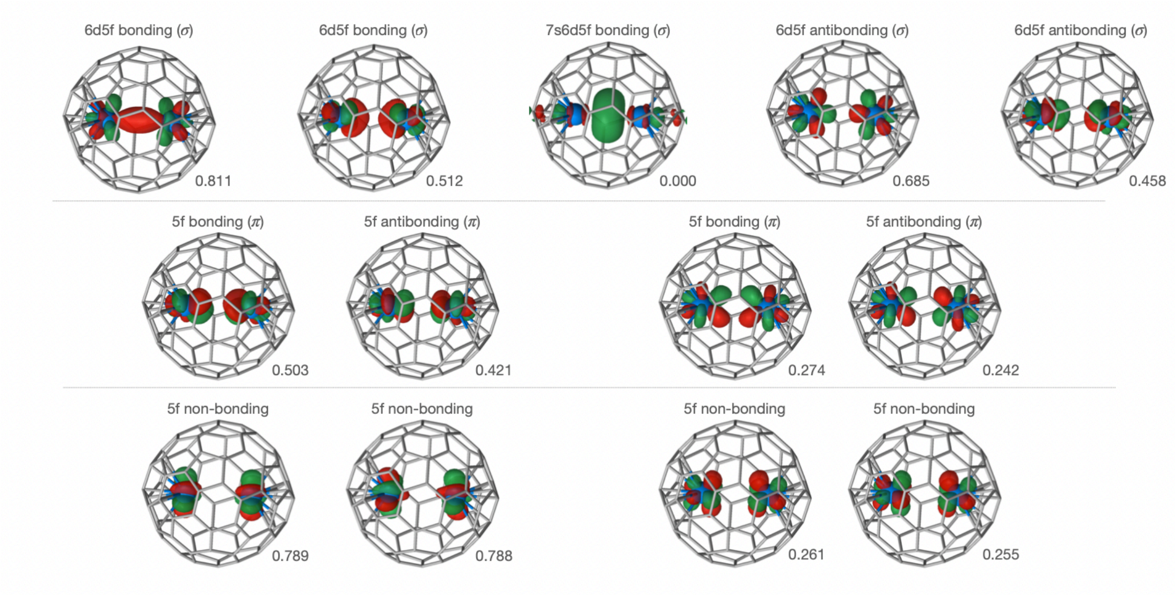

Figure S14. Natural orbitals and natural occupation numbers of the 3rd triplet CASSCF root of A_{1g} symmetry. ΔE(CASSCF) = 6.3 kcal/mol, ΔE(CASPT2) = -1.7 kcal/mol. Estimated BO: sigma + pi contribution = 0.15.

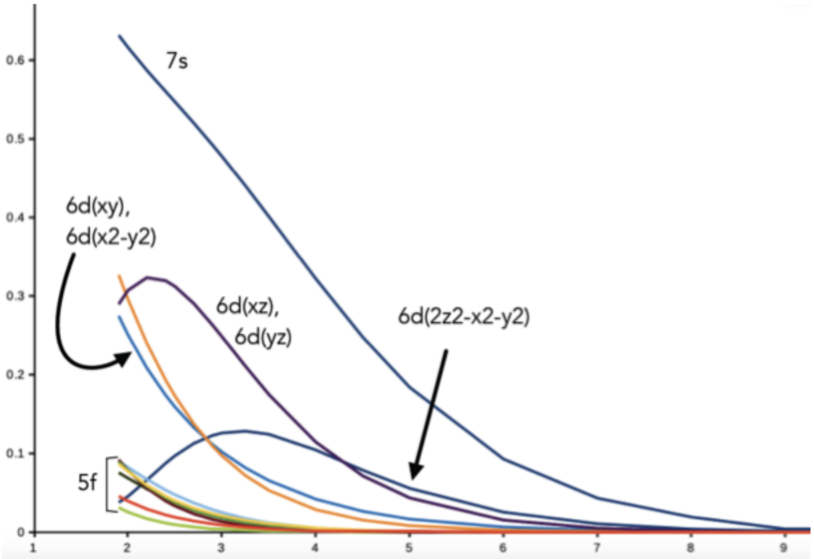

Figure S15. Atomic orbital overlap (U-U). While hybrid 7s/6d orbitals can retain the long-range interactions, 5f orbitals can form strong metal-metal bonds in diuranium EMFs.

Table S1. Average uranium-uranium distances ($d_{U\text{-}U}$) and carbon-uranium distances ($d_{U\text{-}C}$) for the different CPMD simulations.

**Fullerene**	**$d_{U\text{-}U}$ [a]**	**$d_{U\text{-}C}$ [a]**	**Time [b]**
$^{5}C_{78}$	2.47	2.46	50.0
$^{1}C_{60}$	2.41	2.50	18.3
$^{271}C_{50}$	2.48	2.41	10.8
$^{262}C_{50}$	2.58	2.43	15.6
$^{169}C_{48}$	2.46	2.41	36.5
$^{109}C_{46}$	2.50	2.36	18.9
$^{75}C_{44}$	2.44	2.35	16.8

[a] Distances in Å. [b] Time in ps.

Table S2. Relative energies (kcal·mol^{-1}), U-U distances (Å), average C-U distances (Å), U spin densities (SD) and S^2 value for several diuranium endohedral fullerenes at GE PBE and GGA hybrid PBE0 levels.

**Cage**	**State**	**GGA PBE**						**Hybrid PBE0**					
		**ΔE**	**$d_{U\text{-}U}$**	**$d_{C\text{-}U}$**	**SD U[a]**	**SD U[b]**	**S^2**	**ΔE**	**$d_{U\text{-}U}$**	**$d_{C\text{-}U}$**	**SD U[a]**	**SD U[b]**	**S^2**
C_2(263)-C_{50}	S	10.8	2.503	2.39				8.4	2.464	2.49			
	T	10.8	2.491	2.40	0.79	0.78	2.02	10.2	2.428	2.52	0.66	0.66	2.03
C_s(262)-C_{50}	S	3.8	2.644	2.40				4.0	2.528	2.52			
	T	0.0	2.592	2.42	0.76	0.76	2.02	0.0	2.487	2.55	0.61	0.61	2.02
C_2(260)-C_{50}	S	5.2	2.654	2.39				6.4	2.535	2.43			
	T	2.8	2.622	2.41	0.88	0.78	2.02	5.0	2.475	2.51	0.67	0.65	2.03

Table S3. Atomic orbital contributions (in %) at PBE0/TZP level for $U_2@I_h(7)$-C_{80} and $Th_2@I_h(7)$-C_{80}.

	U-U		Th-Th
	σ_1	σ_2	σ
7s	18.8	23.3	55.2
7p	10.9	12.8	24.2
6d	29.0	29.8	4.7
5f	38.7	31.7	2.0

σ_1

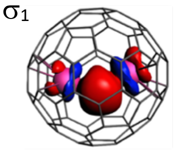

σ_2

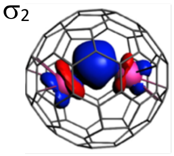

Table S4. Relative CASSCF and CASPT2 energies (in kcal·mol^{-1}) for $U_2@C_{80}$.[a)]

State (config.)[b)]	CASSCF [c)]	CASPT2 [c)]	PBE0[e)]
**Long**			
Singlet ($5f^6$)	0.0	0.0	26.1
singlet ($5f^5(7s/6d)^1$)	5.1	0.0	
Triplet ($5f^6$)	0.0	0.5	17.6
Triplet ($5f^5(7s/6d)^1$)	6.3	-1.3	
Quintuplet ($5f^6$)	0.1	1.1	10.0
Septuplet ($5f^6$)	0.2	1.7	0.0
**Short**			
Singlet ($5f^6$)	0.0	0.0<br>(22.7)[d)]	9.4
Triplet ($5f^6$)	11.2	38.2	7.9

a) Single point calculations at CASSCF and CASPT2 levels were performed using the PBE0 geometry for long ($d_{U\text{-}U}$=3.793 Å) and short bond lengths ($d_{U\text{-}U}$ =2.406 Å). b) The dominant electronic configuration is given in parentheses; c) For the long arrangement, the singlet, triplet, quintet and septuplet states dominated by the $5f^6$ electronic configuration lie within a range of less than 2 kcal mol^{-1}. None of these states exhibits a significant U-U interaction. However, there are several other states that are close in energy, some are dominated by excited $5f^6$ electronic configurations in which higher-lying 5f orbitals become partially occupied and some states have significant contributions from $5f^5(7s/6d)^1$ configuration leading to a direct metal-metal overlap. In the short arrangement, these states are well separated (>8 kcal/mol) from the singlet($5f^6$) state; d) Value in parenthesis corresponds to the relative energy of the singlet state with respect to the lowest energy states for the long arrangement; e) PBE0 energies correspond to relaxed structures for each state. Both CASPT2 and PBE0 predict the long arrangement to be the most stable form.

$U_2@C_s(263)$-C_{50}

Bond Energy -12918.58 kcal/mol

C	3.193541	-2.028094	-1.425218
C	3.584298	-2.146660	-0.012257
C	4.202013	-0.894141	0.375639
C	4.212067	-0.022070	-0.792773
C	3.535902	-0.678166	-1.933348
C	1.859855	-2.572116	-1.536898
C	1.421733	-3.009098	-0.231340
C	2.443848	-2.665308	0.724089
C	1.975857	-1.981248	1.930292
C	2.574443	-0.705796	2.296996
C	3.689064	-0.139854	1.520558
C	3.522935	1.239300	1.074352
C	3.818824	1.304821	-0.348683
C	2.947550	2.076891	-1.174248
C	2.323017	1.499798	-2.349956
C	2.459607	0.067454	-2.600746
C	1.177608	-0.595434	-2.856558
C	0.863551	-1.903810	-2.320361
C	-0.559322	-2.007195	-2.070674
C	-1.024825	-2.679921	-0.857256
C	-0.001544	-3.009715	0.101803
C	-0.441335	-2.560263	1.402549
C	0.552608	-1.880196	2.178927
C	0.233811	-0.567009	2.700474
C	1.498035	0.151082	2.730933
C	1.539535	1.570534	2.561780
C	2.500252	2.081753	1.618407
C	1.810642	3.052993	0.790152
C	2.029345	3.054908	-0.608454
C	0.923381	3.175805	-1.500510
C	1.092647	2.222931	-2.569052
C	-0.135770	1.538772	-2.741130
C	-0.089176	0.117737	-2.895019
C	-1.162506	-0.738158	-2.451552
C	-2.279140	-0.167813	-1.681256
C	-2.789337	-0.911425	-0.528149
C	-2.167163	-2.157432	-0.126631
C	-1.776925	-2.022162	1.284949
C	-2.124192	-0.668058	1.778358
C	-1.050547	0.088544	2.437572
C	-0.919036	1.518531	2.171050
C	0.308701	2.248421	2.382297
C	0.474569	3.190279	1.303515
C	-0.630967	3.055775	0.412827
C	-0.412216	3.039352	-0.985685
C	-1.098382	2.056839	-1.803428
C	-2.117995	1.216672	-1.250112
C	-2.414104	1.296591	0.172128
C	-2.802654	-0.026779	0.630678
C	-1.545656	2.080701	0.989247
U	-0.522813	-0.409181	0.018839
U	1.933649	-0.406203	-0.176533

$U_2@C_s(262)$-C_{50}

Bond Energy -12927.00 kcal/mol

S^2 2.02197

C	2.645885	-1.080424	-2.027180
C	2.660384	-2.125554	-1.022012
C	3.274867	-1.605668	0.182835
C	3.687373	-0.243814	-0.094084
C	3.302614	0.087588	-1.479006
C	1.287578	-1.021107	-2.599541
C	0.554570	-2.118798	-2.013075
C	1.349935	-2.724851	-0.976935
C	0.708698	-2.966986	0.297315
C	1.270653	-2.345379	1.480854
C	2.540209	-1.619556	1.435891
C	2.569017	-0.269832	1.980562
C	3.285571	0.587623	1.040073
C	2.753578	1.880440	0.750496
C	2.627952	2.312817	-0.628225
C	2.632577	1.322251	-1.680071
C	1.326149	1.407259	-2.364249
C	0.576445	0.237324	-2.754199
C	-0.913064	0.265096	-2.648423
C	-1.641066	-0.966837	-2.391266
C	-0.873150	-2.092374	-1.911704
C	-1.535243	-2.671191	-0.771481
C	-0.729215	-2.940081	0.399761
C	-1.094096	-2.301052	1.649423
C	0.145054	-1.837339	2.235831
C	0.213149	-0.550294	2.853366
C	1.403059	0.256032	2.672821
C	0.953265	1.601451	2.443619
C	1.613359	2.423203	1.480376
C	0.831685	3.217230	0.576192
C	1.433289	3.080335	-0.728930
C	0.643934	2.555469	-1.804740
C	-0.758513	2.581720	-1.704517
C	-1.555256	1.461135	-2.158104
C	-2.753377	1.422895	-1.295315
C	-3.433087	0.212770	-0.998656
C	-2.905345	-0.977325	-1.631483
C	-2.816419	-2.024595	-0.631857
C	-3.233589	-1.484800	0.647030
C	-2.329606	-1.529222	1.783555
C	-2.230122	-0.180694	2.322940
C	-0.959410	0.299881	2.842122
C	-0.497242	1.628639	2.547515
C	-1.256019	2.476522	1.685203
C	-0.581882	3.243936	0.677220
C	-1.367154	3.132872	-0.528921
C	-2.563510	2.410311	-0.257616
C	-2.508045	1.979022	1.126163
C	-3.040382	0.705476	1.491247
C	-3.629109	-0.107654	0.427839
U	-1.231186	-0.250962	-0.001842
U	1.248952	-0.294319	-0.178031

$U_2@C_s(263)-C_{50}$

Bond Energy -12918.58 kcal/mol

C	3.193541	-2.028094	-1.425218
C	3.584298	-2.146660	-0.012257
C	4.202013	-0.894141	0.375639
C	4.212067	-0.022070	-0.792773
C	3.535902	-0.678166	-1.933348
C	1.859855	-2.572116	-1.536898
C	1.421733	-3.009098	-0.231340
C	2.443848	-2.665308	0.724089
C	1.975857	-1.981248	1.930292
C	2.574443	-0.705796	2.296996
C	3.689064	-0.139854	1.520558
C	3.522935	1.239300	1.074352
C	3.818824	1.304821	-0.348683
C	2.947550	2.076891	-1.174248
C	2.323017	1.499798	-2.349956
C	2.459607	0.067454	-2.600746
C	1.177608	-0.595434	-2.856558
C	0.863551	-1.903810	-2.320361
C	-0.559322	-2.007195	-2.070674
C	-1.024825	-2.679921	-0.857256
C	-0.001544	-3.009715	0.101803
C	-0.441335	-2.560263	1.402549
C	0.552608	-1.880196	2.178927
C	0.233811	-0.567009	2.700474
C	1.498035	0.151082	2.730933
C	1.539535	1.570534	2.561780
C	2.500252	2.081753	1.618407
C	1.810642	3.052993	0.790152
C	2.029345	3.054908	-0.608454
C	0.923381	3.175805	-1.500510
C	1.092647	2.222931	-2.569052
C	-0.135770	1.538772	-2.741130
C	-0.089176	0.117737	-2.895019
C	-1.162506	-0.738158	-2.451552
C	-2.279140	-0.167813	-1.681256
C	-2.789337	-0.911425	-0.528149
C	-2.167163	-2.157432	-0.126631
C	-1.776925	-2.022162	1.284949
C	-2.124192	-0.668058	1.778358
C	-1.050547	0.088544	2.437572
C	-0.919036	1.518531	2.171050
C	0.308701	2.248421	2.382297
C	0.474569	3.190279	1.303515
C	-0.630967	3.055775	0.412827
C	-0.412216	3.039352	-0.985685
C	-1.098382	2.056839	-1.803428
C	-2.117995	1.216672	-1.250112
C	-2.414104	1.296591	0.172128
C	-2.802654	-0.026779	0.630678
C	-1.545656	2.080701	0.989247
U	-0.522813	-0.409181	0.018839
U	1.933649	-0.406203	-0.176533

$U_2@C_2(260)-C_{50}$

Bond Energy -12922.05 kcal/mol

S^2 2.03193

C	2.680457	-1.348652	-1.078500
C	2.711881	-1.811562	0.277024
C	2.936612	-0.679193	1.105537
C	3.164201	0.485897	0.289496
C	2.972196	0.056020	-1.110336
C	1.532693	-1.977253	-1.720261
C	0.974750	-2.926857	-0.771497
C	1.617517	-2.695663	0.503493
C	0.813702	-2.512383	1.664786

$U_2@C_s(262)-C_{50}$

Bond Energy -12927.00 kcal/mol

S^2 2.02197

C	2.645885	-1.080424	-2.027180
C	2.660384	-2.125554	-1.022012
C	3.274867	-1.605668	0.182835
C	3.687373	-0.243814	-0.094084
C	3.302614	0.087588	-1.479006
C	1.287578	-1.021107	-2.599541
C	0.554570	-2.118798	-2.013075
C	1.349935	-2.724851	-0.976935
C	0.708698	-2.966986	0.297315
C	1.270653	-2.345379	1.480854
C	2.540209	-1.619556	1.435891
C	2.569017	-0.269832	1.980562
C	3.285571	0.587623	1.040073
C	2.753578	1.880440	0.750496
C	2.627952	2.312817	-0.628225
C	2.632577	1.322251	-1.680071
C	1.326149	1.407259	-2.364249
C	0.576445	0.237324	-2.754199
C	-0.913064	0.265096	-2.648423
C	-1.641066	-0.966837	-2.391266
C	-0.873150	-2.092374	-1.911704
C	-1.535243	-2.671191	-0.771481
C	-0.729215	-2.940081	0.399761
C	-1.094096	-2.301052	1.649423
C	0.145054	-1.837339	2.235831
C	0.213149	-0.550294	2.853366
C	1.403059	0.256032	2.672821
C	0.953265	1.601451	2.443619
C	1.613359	2.423203	1.480376
C	0.831685	3.217230	0.576192
C	1.433289	3.080335	-0.728930
C	0.643934	2.555469	-1.804740
C	-0.758513	2.581720	-1.704517
C	-1.555256	1.461135	-2.158104
C	-2.753377	1.422895	-1.295315
C	-3.433087	0.212770	-0.998656
C	-2.905345	-0.977325	-1.631483
C	-2.816419	-2.024595	-0.631857
C	-3.233589	-1.484800	0.647030
C	-2.329606	-1.529222	1.783555
C	-2.230122	-0.180694	2.322940
C	-0.959410	0.299881	2.842122
C	-0.497242	1.628639	2.547515
C	-1.256019	2.476522	1.685203
C	-0.581882	3.243936	0.677220
C	-1.367154	3.132872	-0.528921
C	-2.563510	2.410311	-0.257616
C	-2.508045	1.979022	1.126163
C	-3.040382	0.705476	1.491247
C	-3.629109	-0.107654	0.427839
U	-1.231186	-0.250962	-0.001842
U	1.248952	-0.294319	-0.178031

$U_2@C_1(196)-C_{48}$

Bond Energy -12393.91 kcal/mol

C	-3.009240	-1.891564	-0.402350
C	-2.973014	-1.001237	-1.576840
C	-3.227447	0.369508	-1.133178
C	-3.440501	0.336004	0.308912
C	-3.302211	-1.064590	0.788703
C	-1.829456	-2.776470	-0.474739
C	-1.127329	-2.441704	-1.715966
C	-1.761659	-1.273897	-2.320148
C	-0.879439	-0.170324	-2.729936

C 1.008960 -1.340136 2.495012
C 2.031867 -0.376031 2.165962
C 1.763794 1.052563 2.083805
C 2.479756 1.615672 0.928580
C 1.763625 2.502927 0.030918
C 1.722252 2.139539 -1.403490
C 2.176769 0.870838 -1.971435
C 1.001766 0.241551 -2.634152
C 0.670990 -1.177144 -2.531291
C -0.725110 -1.560584 -2.586884
C -1.181863 -2.708639 -1.870886
C -0.390645 -3.260649 -0.813165
C -1.195993 -3.244119 0.381008
C -0.608622 -2.795443 1.606365
C -1.297152 -1.840254 2.426208
C -0.304670 -0.907400 2.922869
C -0.584147 0.488551 2.859572
C 0.410306 1.451198 2.416454
C -0.265494 2.410848 1.602460
C 0.375961 2.872766 0.388833
C -0.441233 2.821256 -0.787614
C 0.359960 2.303866 -1.838802
C -0.104413 1.170991 -2.582159
C -1.514112 0.788513 -2.544440
C -1.826642 -0.612846 -2.644368
C -2.976506 -1.222061 -1.935396
C -2.516663 -2.485865 -1.412501
C -2.529323 -2.778548 0.010713
C -3.289121 -1.939450 0.876737
C -2.629262 -1.398058 2.064914
C -2.970904 0.006425 2.173786
C -1.897331 0.952584 2.463098
C -1.688352 2.125508 1.642951
C -2.512514 2.307029 0.475292
C -1.835222 2.492407 -0.771651
C -2.419233 1.594936 -1.758816
C -3.604560 0.991834 -1.128700
C -3.869586 -0.436395 -1.149361
C -4.053991 -0.851829 0.259741
C -3.894605 0.316543 1.102721
C -3.645199 1.446904 0.246509
U -1.736807 -0.130356 0.000996
U 0.690226 0.348480 -0.085646

U_2@C_1(103)-C_{46}
Bond Energy -11899.51 kcal/mol

C 2.451342 -2.500278 0.563570
C 2.082399 -2.755836 -0.841377
C 0.727740 -3.276681 -0.842570
C 0.249127 -3.316428 0.536134
C 1.303534 -2.788446 1.431159
C 3.090464 -1.188831 0.597714
C 3.059705 -0.645876 -0.753758
C 2.371968 -1.577271 -1.636481
C 1.363654 -0.991710 -2.509372
C 0.034447 -1.575663 -2.583000
C -0.283212 -2.648213 -1.659812
C -1.447975 -2.426116 -0.835065
C -1.107772 -2.766065 0.519135
C -1.555215 -1.883919 1.550096
C -0.536522 -1.443590 2.472635
C 0.883004 -1.762804 2.366121
C 1.572578 -0.425322 2.436419
C 2.645565 -0.143546 1.487908
C 2.517029 1.071346 0.706167
C 2.698902 0.743639 -0.700565
C 1.851606 1.366499 -1.717200
C 1.173956 0.450676 -2.646918

C -1.118307 1.178640 -2.277840
C -2.258661 1.424304 -1.422211
C -1.949295 2.133487 -0.201515
C -2.634472 1.452672 0.871584
C -1.885609 1.250159 2.062179
C -1.882434 -0.074677 2.607280
C -2.410504 -1.249953 1.906843
C -1.298489 -2.239312 1.896862
C -0.963726 -3.017901 0.703261
C 0.423851 -3.336376 0.505406
C 1.027524 -3.303232 -0.812679
C 0.283797 -2.634492 -1.830420
C 1.125153 -1.550501 -2.301101
C 0.560906 -0.326340 -2.732144
C 1.226177 0.933921 -2.448727
C 0.174292 1.860482 -2.104439
C 0.388572 2.802141 -1.010299
C -0.637135 2.764363 0.006660
C 0.029872 2.698861 1.290168
C -0.536095 1.792839 2.227283
C 0.310694 0.791166 2.837700
C -0.565354 -0.338863 3.054029
C -0.214343 -1.664000 2.666735
C 1.147961 -1.884597 2.380019
C 1.450838 -2.864136 1.417116
C 2.609816 -2.441160 0.679004
C 2.319712 -2.686979 -0.711810
C 2.395930 -1.590820 -1.610359
C 2.993841 -0.329945 -1.206156
C 2.536688 0.946620 -1.743353
C 2.764710 1.951672 -0.706095
C 1.694646 2.869869 -0.341469
C 1.475213 2.739220 1.097372
C 2.373993 1.704063 1.672763
C 1.722047 0.628812 2.462461
C 2.087690 -0.760648 2.224890
C 2.994513 -1.159692 1.130375
C 3.385511 -0.134387 0.189126
C 3.210605 1.269197 0.517259
U -1.211397 -0.412208 -0.092819
U 0.971192 0.645862 0.080400

U_2@D_2(75)-C_{44}
Bond Energy -11402.14 kcal/mol

C 3.436920 0.655773 -0.495331
C 3.436071 -0.519315 0.391486
C 2.874984 -0.101721 1.679622
C 2.520695 1.311789 1.567247
C 2.854236 1.818809 0.212759
C 2.874882 0.238548 -1.783336
C 2.519000 -1.174544 -1.670343
C 2.852093 -1.681237 -0.316107
C 1.734104 -2.395355 0.326517
C 1.195315 -2.019904 1.631218
C 1.708146 -0.795186 2.246036
C 0.718134 0.199008 2.587983
C 1.207847 1.492050 2.173231
C 0.235205 2.371709 1.590409
C 0.596823 2.987270 0.340386
C 1.736627 2.532817 -0.430242
C 1.197192 2.158256 -1.735681
C 1.708847 0.932590 -2.350386
C 0.718141 -0.061292 -2.691470
C 1.206571 -1.354639 -2.276514
C 0.233218 -2.234392 -1.694173
C 0.594594 -2.850149 -0.444032

C	-0.203635	0.713413	-3.084728
C	-0.920200	-0.540580	-2.931231
C	-2.242869	-0.509263	-2.287877
C	-2.370000	-1.352681	-1.126547
C	-2.946390	-0.570550	-0.058195
C	-2.384254	-0.746330	1.237286
C	-1.868978	0.436613	1.917901
C	-0.717611	-0.047248	2.660171
C	0.562671	0.582025	2.617208
C	0.571902	1.864989	1.974948
C	1.652628	2.148943	1.072871
C	1.132343	2.956506	0.026402
C	1.199273	2.575392	-1.387683
C	-0.110571	2.897995	-1.959548
C	-0.871969	1.999903	-2.814336
C	-2.228523	1.999417	-2.249178
C	-2.893487	0.748900	-1.971406
C	-3.260033	0.741969	-0.555971
C	-2.794301	1.984225	0.133026
C	-1.939092	1.771729	1.339639
C	-0.654636	2.496383	1.466490
C	-0.220715	3.303253	0.332423
C	-0.955669	3.379297	-0.889487
C	-2.246851	2.776972	-1.002997
U	-0.840268	0.873929	-0.686423
U	0.707976	-0.971271	0.016648

C	-0.601881	-2.849202	0.343411
C	-0.240375	-2.232332	1.592850
C	-1.212967	-1.351888	2.174606
C	-0.723335	-0.058407	2.588101
C	-1.713385	0.935467	2.245211
C	-1.200508	2.159466	1.628993
C	-1.739283	2.533747	0.323898
C	-0.599647	2.987484	-0.447083
C	-0.238159	2.370757	-1.696717
C	-1.211591	1.490477	-2.278228
C	-0.723091	0.196682	-2.691671
C	-1.713759	-0.796862	-2.349469
C	-1.202089	-2.021779	-1.733366
C	-1.741570	-2.395198	-0.427725
C	-2.859119	-1.680450	0.214386
C	-2.525702	-1.172130	1.568305
C	-2.880117	0.241443	1.679311
C	-3.441204	0.657780	0.390734
C	-2.857299	1.819047	-0.318084
C	-2.524056	1.311004	-1.671804
C	-2.879831	-0.102229	-1.783287
C	-3.441813	-0.518181	-0.494891
U	-1.232212	0.067833	-0.051716
U	1.227298	0.070848	-0.051598

U_2@C_{60} orientation O1

Bond Energy -15450.63 kcal/mol

S^2 6.08564

C	-3.044606	-0.923277	1.581948
C	-3.508425	-0.665382	0.249601
C	-3.508425	0.665382	-0.249601
C	-3.063330	1.768614	0.572908
C	-2.568553	1.482955	1.866261
C	-2.570371	0.142843	2.389900
C	-3.063330	-1.768614	-0.572908
C	-3.044606	0.923277	-1.581948
C	-2.330450	2.705694	-0.249601
C	-1.408892	2.154585	2.389900
C	-1.446392	-0.005739	3.292746
C	-2.321885	-2.175067	1.581948
C	0.722721	3.098345	1.581948
C	1.446392	-0.005739	3.292746
C	-0.722721	3.098345	1.581948
C	-1.177975	3.371076	0.249601
C	0.000000	3.537228	-0.572908
C	-1.161480	2.297429	-2.389900
C	-2.321885	2.175067	-1.581948
C	-2.568553	-1.482955	-1.866261
C	-1.408892	-2.154585	-2.389900
C	0.718226	-1.255482	-3.292746
C	-1.446392	0.005739	-3.292746
C	-2.570371	-0.142843	-2.389900
C	0.728166	-1.249743	3.292746
C	-0.728166	-1.249743	3.292746
C	-2.330450	-2.705694	0.249601
C	-1.161480	-2.297429	2.389900
C	0.000000	-2.965910	1.866261
C	-0.718226	1.255482	3.292746
C	-1.177975	-3.371076	-0.249601
C	0.718226	1.255482	3.292746
C	-0.718226	-1.255482	-3.292746
C	0.722721	-3.098345	-1.581948
C	0.000000	-3.537228	0.572908
C	-0.722721	-3.098345	-1.581948
C	0.728166	1.249743	-3.292746
C	0.000000	2.965910	-1.866261
C	-0.728166	1.249743	-3.292746

U_2@C_{60} orientation O2

Bond Energy -15462.40 kcal/mol

C	-2.948772	-0.807302	1.540231
C	-3.393958	-0.515079	0.207492
C	-3.408218	0.851263	-0.227404
C	-3.075421	1.980347	0.650256
C	-2.611407	1.665893	1.998975
C	-2.533648	0.266755	2.388539
C	-2.991803	-1.599958	-0.649933
C	-2.915979	1.122274	-1.539966
C	-2.311503	2.919810	-0.176340
C	-1.390632	2.307026	2.494798
C	-1.338684	0.056163	3.171451
C	-2.246099	-2.067091	1.510087
C	0.789819	3.154047	1.634267
C	1.506940	0.032716	3.205565
C	-0.657535	3.228157	1.672456
C	-1.113836	3.553689	0.331667
C	0.051064	3.644291	-0.516954
C	-1.107540	2.565092	-2.390076
C	-2.232807	2.376140	-1.521074
C	-2.504023	-1.309638	-1.967073
C	-1.397764	-2.015953	-2.588872
C	0.782215	-1.061868	-3.444742
C	-1.360216	0.219929	-3.283554
C	-2.475084	0.048235	-2.408553
C	0.801033	-1.185217	3.177532
C	-0.647747	-1.177151	3.158103
C	-2.288970	-2.558047	0.154549
C	-1.105272	-2.262918	2.328116
C	0.054812	-2.917322	1.797107
C	-0.619761	1.304250	3.212462
C	-1.117574	-3.160533	-0.390123
C	0.798442	1.282242	3.227623
C	-0.678532	-1.070332	-3.448223
C	0.771324	-2.931165	-1.749708
C	0.046692	-3.329350	0.446634
C	-0.681604	-2.953392	-1.751137
C	0.767093	1.468009	-3.231868
C	0.059167	3.226958	-1.880282
C	-0.667760	1.474082	-3.221025

C	3.044606	0.923277	-1.581948
C	1.408892	2.154585	2.389900
C	1.446392	0.005739	-3.292746
C	2.570371	0.142843	2.389900
C	1.408892	-2.154585	-2.389900
C	1.161480	-2.297429	2.389900
C	3.063330	1.768614	0.572908
C	1.161480	2.297429	-2.389900
C	2.321885	2.175067	-1.581948
C	2.330450	2.705694	-0.249601
C	1.177975	3.371076	0.249601
C	2.568553	-1.482955	-1.866261
C	2.321885	-2.175067	1.581948
C	1.177975	-3.371076	-0.249601
C	3.044606	-0.923277	1.581948
C	3.508425	0.665382	-0.249601
C	2.330450	-2.705694	0.249601
C	3.063330	-1.768614	-0.572908
C	3.508425	-0.665382	0.249601
C	2.570371	-0.142843	-2.389900
C	2.568553	1.482955	1.866261
U	0.000000	0.000000	-1.226341
U	0.000000	0.000000	1.226341

$U_2@C_{80}$ long

Bond Energy -20523.00 kcal/mol

S^2 12.07597

C	-0.353456	-4.645479	-2.581569
C	-0.259773	-3.410852	-3.304934
C	-1.546039	-5.076134	-1.920455
C	0.765546	-5.181354	-1.877791
C	-1.426575	-2.605279	-3.353675
C	1.010848	-2.775681	-3.347297
C	-2.681061	-4.198399	-1.903474
C	-1.192029	-5.953316	-0.807849
C	0.273773	-5.965969	-0.782003
C	2.142876	-3.313795	-2.636154
C	2.028000	-4.500273	-1.863340
C	-2.624233	-2.994279	-2.651454
C	-1.336050	-1.177442	-3.324711
C	1.101316	-1.348146	-3.306749
C	-3.461195	-4.219163	-0.708939
C	1.031065	-5.992338	0.437214
C	-1.986556	-5.981804	0.417356
C	2.803812	-4.620174	-0.680459
C	-0.066277	-0.545378	-3.235398
C	2.930232	-2.215658	-2.159399
C	-3.270832	-1.793135	-2.190579
C	-3.108336	-5.056839	0.431864
C	2.289511	-0.993662	-2.570965
C	2.294874	-5.340712	0.461118
C	-2.477608	-0.673561	-2.603801
C	-1.208082	-6.011126	1.652576
C	0.257881	-6.023775	1.646028
C	-4.067884	-3.011686	-0.241536
C	3.598266	-3.529015	-0.207725
C	3.615160	-2.293831	-0.916740
C	-3.972224	-1.774275	-0.954788
C	0.017282	0.653322	-2.452848
C	2.335588	0.172981	-1.765248
C	-3.476042	-4.274427	1.606504
C	-2.373384	0.474622	-1.773683
C	2.788412	-4.675875	1.642527
C	-4.077246	-3.046036	1.189571
C	3.588781	-3.563651	1.233210
C	-1.577034	-5.188703	2.801438
C	1.200751	1.050731	-1.755683
C	-1.111172	1.155410	-1.739207
C	0.734895	-5.293533	2.785076

C	3.068489	1.107452	-1.613282
C	1.520312	2.233752	2.419857
C	1.459343	0.217946	-3.302961
C	2.681387	0.214723	2.370448
C	1.493136	-2.009291	-2.581569
C	1.239244	-2.260856	2.328281
C	3.124065	1.883158	0.581829
C	1.224375	2.544092	-2.386191
C	2.383000	2.374940	-1.587317
C	2.395323	2.843167	-0.232277
C	1.228887	3.441376	0.292788
C	2.610155	-1.305765	-1.971378
C	2.367012	-2.075632	1.510389
C	1.209120	-3.156410	-0.386313
C	3.102607	-0.817965	1.538252
C	3.546281	0.809883	-0.278336
C	2.359069	-2.534540	0.150228
C	3.076850	-1.582569	-0.660569
C	3.541421	-0.519351	0.186130
C	2.604896	0.057256	-2.438728
C	2.685315	1.588590	1.884168
U	0.034494	-0.495884	-1.192518
U	-0.879557	0.950104	0.432154

$U_2@C_{80}$ short

Bond Energy -20476.60 kcal/mol

C	-47.032988	4.067191	27.517387
C	-47.203102	5.498571	27.489735
C	-46.477061	6.060243	28.585219
C	-45.873302	4.986915	29.331030
C	-46.226815	3.714821	28.685348
C	-45.221600	2.673590	28.561652
C	-45.148848	1.996741	27.303731
C	-46.027307	2.288755	26.185940
C	-46.950609	3.370975	26.255590
C	-47.203535	4.080092	25.055084
C	-47.445141	5.507842	25.062139
C	-47.406977	6.231502	26.272726
C	-46.923400	7.574931	26.233352
C	-46.204493	8.142875	27.338279
C	-45.944923	7.409534	28.519577
C	-44.735113	7.703553	29.239172
C	-44.034598	6.583998	29.812260
C	-44.565388	5.234232	29.875593
C	-43.627304	4.176220	29.845321
C	-43.940024	2.926534	29.162753
C	-42.706128	2.447240	28.600682
C	-42.657913	1.724537	27.369198
C	-43.918083	1.490695	26.740067
C	-44.039354	1.465638	25.325127
C	-45.341329	1.973908	24.981448
C	-45.552580	2.723391	23.779326
C	-46.531629	3.758263	23.829705
C	-46.357317	4.973414	23.085022
C	-46.924274	6.050132	23.842405
C	-46.345516	7.355953	23.812258
C	-46.390371	8.115169	25.024185
C	-45.327289	9.010890	25.372478
C	-45.210490	9.066458	26.804654
C	-43.968208	9.338004	27.476178
C	-43.743885	8.670214	28.746384
C	-42.435034	8.076804	29.016180
C	-42.629930	6.820461	29.695550
C	-41.693259	5.736959	29.604773
C	-42.217469	4.413184	29.716907
C	-41.648653	3.345612	28.959095
C	-40.528930	3.547408	28.091476
C	-40.460520	2.771365	26.905706

C	3.700689	-1.091975	-0.143819
C	-3.965074	-0.574364	-0.187427
C	3.099794	0.136601	-0.560777
C	-2.711879	-4.310921	2.810969
C	-3.164813	0.537908	-0.596777
C	1.997031	-4.612640	2.819485
C	-0.393572	-4.791395	3.498688
C	-3.991580	-1.844198	1.962509
C	3.595892	-2.363765	2.000579
C	3.691241	-1.126356	1.287245
C	-3.974645	-0.608982	1.253526
C	0.831820	1.873222	-0.606790
C	-0.634167	1.885535	-0.600190
C	-2.665840	-3.144333	3.616757
C	2.101243	-3.464441	3.649588
C	2.732385	0.919279	0.613866
C	-2.671303	1.202741	0.584665
C	-3.306617	-1.922366	3.205186
C	2.894506	-2.344896	3.236341
C	-0.310066	-3.592675	4.281242
C	3.084718	0.081229	1.754583
C	1.610383	1.844196	0.628420
C	-1.407470	1.854156	0.608565
C	-3.180194	0.482177	1.726239
C	0.959706	-2.960580	4.370540
C	-1.477671	-2.789892	4.352599
C	-2.519214	-0.824245	3.681876
C	2.247833	-1.143768	3.697126
C	2.304565	0.060328	2.949129
C	0.815639	1.815075	1.853452
C	-0.650126	1.827671	1.827686
C	-2.404357	0.362269	2.909113
C	1.050206	-1.532744	4.399420
C	-1.387195	-1.362359	4.393062
C	1.169640	0.938037	2.966217
C	-1.141930	1.043356	2.923614
C	-0.116590	-0.727161	4.350724
C	-0.022917	0.507483	3.627409
U	-0.861492	-3.885989	0.471122
U	0.487689	-0.252114	0.573640

C	-41.517796	1.853084	26.550563
C	-41.640641	1.849188	25.116533
C	-42.906911	1.693011	24.481812
C	-43.137628	2.374636	23.255822
C	-44.445588	2.881988	22.910655
C	-44.266213	4.098488	22.168816
C	-45.192631	5.172484	22.282163
C	-44.679608	6.501111	22.181692
C	-45.256004	7.577628	22.930841
C	-44.191308	8.492484	23.272571
C	-44.201372	9.171587	24.507467
C	-42.952422	9.410476	25.156747
C	-42.844345	9.441208	26.597476
C	-41.523161	8.937772	26.924970
C	-41.308812	8.195021	28.121157
C	-40.353430	7.149943	28.067957
C	-40.536584	5.925564	28.818933
C	-39.953123	4.854641	28.066931
C	-39.417304	5.403571	26.856036
C	-39.448725	4.662068	25.635464
C	-39.936661	3.321307	25.690795
C	-40.663842	2.753208	24.589285
C	-40.929338	3.512476	23.409288
C	-42.160960	3.274852	22.725465
C	-42.853990	4.339782	22.053749
C	-42.333509	5.661764	22.046264
C	-43.268040	6.741439	22.061101
C	-42.963846	7.967509	22.734480
C	-41.723761	8.136427	23.415981
C	-41.736611	8.911884	24.617522
C	-40.856349	8.605572	25.714525
C	-39.931451	7.514303	25.642368
C	-39.660531	6.818717	26.856786
C	-39.869389	6.796114	24.423543
C	-40.754138	7.100913	23.322670
C	-41.055048	5.876946	22.646672
C	-40.363591	4.812807	23.321253
C	-39.631291	5.379851	24.420424
U	-44.572642	4.678807	27.226517
U	-43.680653	6.883673	27.134412
